# Improved metagenome assemblies through selective enrichment of bacterial genomic DNA from eukaryotic host genomic DNA using ATAC-seq

**DOI:** 10.3389/fmicb.2024.1352378

**Published:** 2024-02-15

**Authors:** Lindsey J. Cantin, Julie C. Dunning Hotopp, Jeremy M. Foster

**Affiliations:** ^1^Biochemistry and Microbiology Division, New England BioLabs, Ipswich, MA, United States; ^2^Institute for Genome Sciences, University of Maryland School of Medicine, Baltimore, MD, United States

**Keywords:** genome assembly, bacterial symbiont, *Wolbachia*, ATAC-seq, filariasis, epigenetics, metagenome assembled genomes

## Abstract

Genomics can be used to study the complex relationships between hosts and their microbiota. Many bacteria cannot be cultured in the laboratory, making it difficult to obtain adequate amounts of bacterial DNA and to limit host DNA contamination for the construction of metagenome-assembled genomes (MAGs). For example, *Wolbachia* is a genus of exclusively obligate intracellular bacteria that live in a wide range of arthropods and some nematodes. While *Wolbachia* endosymbionts are frequently described as facultative reproductive parasites in arthropods, the bacteria are obligate mutualistic endosymbionts of filarial worms. Here, we achieve 50-fold enrichment of bacterial sequences using ATAC-seq (Assay for Transposase-Accessible Chromatin using sequencing) with *Brugia malayi* nematodes, containing *Wolbachia* (*w*Bm). ATAC-seq uses the Tn5 transposase to cut and attach Illumina sequencing adapters to accessible DNA lacking histones, typically thought to be open chromatin. Bacterial and mitochondrial DNA in the lysates are also cut preferentially since they lack histones, leading to the enrichment of these sequences. The benefits of this include minimal tissue input (<1 mg of tissue), a quick protocol (<4 h), low sequencing costs, less bias, correct assembly of lateral gene transfers and no prior sequence knowledge required. We assembled the *w*Bm genome with as few as 1 million Illumina short paired-end reads with >97% coverage of the published genome, compared to only 12% coverage with the standard gDNA libraries. We found significant bacterial sequence enrichment that facilitated genome assembly in previously published ATAC-seq data sets from human cells infected with *Mycobacterium tuberculosis* and *C. elegans* contaminated with their food source, the OP50 strain of *E. coli*. These results demonstrate the feasibility and benefits of using ATAC-seq to easily obtain bacterial genomes to aid in symbiosis, infectious disease, and microbiome research.

## 1 Introduction

Many symbiotic bacteria remain uncultured and may even be impossible to culture in the absence of the host (Ashton et al., 2003; Hongoh, 2010; Xie et al., 2019; Masson and Lemaitre, 2020). Genomics and metagenomics have long been used to study these bacteria through the analysis of the host and microbiota genome sequences. The creation of what are now called metagenome assembled genomes (MAGs) from host genome sequencing data was demonstrated for *Wolbachia* endosymbionts in the *Drosophila* genome sequencing projects (Salzberg et al., 2005).

Bacterial endosymbionts can live within eukaryotic hosts long-term, resulting in mutualistic, commensal, and parasitic relationships. In mutualistic relationships, both species benefit from their interactions. The microbial symbionts often provide metabolites from pathways absent in the eukaryotic host, while the host provides a nutrient rich environment for their resident bacteria (Douglas, 2014). For example, the human gut microbiome consists of trillions of bacteria, with many of them metabolizing nutrients from food components nondigestible by the host and protecting their host from pathogen invasion (Bäckhed et al., 2005; Bull and Plummer, 2014). Nitrogen fixing bacteria are common symbionts of plants, animals, fungi and protists where they increase the bioavailability of nitrogen for the eukaryotic host (Kneip et al., 2007).

In parasitic relationships, the bacteria will usually benefit at the expense of the eukaryotic host by causing disease or disrupting normal biological processes during their replication in the host (Balloux and van Dorp, 2017). The pathogenic bacterium *Mycobacterium tuberculosis* (*M. tb*) is an intracellular parasite that lives in macrophages in the respiratory system of mammalian hosts. The host provides a safe niche for bacterial replication in a tissue that allows the bacteria to spread through air droplets. The infection can lead to fibrosis and necrosis of the host’s lung tissue (Smith, 2003).

*Wolbachia* endosymbionts are some of the most abundant intracellular bacteria and are present in almost 60% of all arthropod species and some nematode species (Hilgenboecker et al., 2008). *Wolbachia* endosymbionts are maternally transmitted and live in the reproductive tissues of their host. In insects, the bacteria were first studied as reproductive parasites that act through male killing, feminization, parthenogenesis and cytoplasmic incompatibility (Werren, 1997; Chen et al., 2020). More recently, *Wolbachia* has been found to provide protection to arthropods from other pathogens, such as RNA viruses (Hedges et al., 2008; Ye et al., 2013; Drew et al., 2021). This demonstrates the complex relationships between hosts and their symbionts and how there is a continuum between parasitism and mutualism that can often be context dependent.

*Wolbachia* is also present in many filarial nematodes as an obligate mutualistic endosymbiont, meaning the worms cannot survive without the bacteria and the bacteria cannot live outside of the worms (Mclaren et al., 1975; Sironi et al., 1995; Bandi et al., 1998, 2001; Quek et al., 2022). Filarial nematodes are insect-borne parasites that cause filariasis, one of the leading causes of morbidity in the world. *Brugia malayi* (*B. malayi*) and *Wuchereria bancrofti* are the predominant species that cause lymphatic filariasis, which can lead to elephantiasis and disfigurement. Onchocerciasis, caused by *Onchocerca volvulus*, can result in visual impairment (WHO, 1995; WHO, 2019; Medeiros et al., 2021). Various combinations of ivermectin, diethylcarbamazine, and albendazole have been used to prevent filarial infections, but these drugs cannot treat established infections (Campbell, 1991; Richards et al., 2001; Mackenzie et al., 2002; Molyneux et al., 2003). Antibiotics, such as doxycycline and rifampicin, kill the endosymbiotic bacteria, leading to the eventual death of the adult worms (Taylor et al., 2005; Bazzocchi et al., 2008; Hoerauf et al., 2008; Specht et al., 2008; Supali et al., 2008; Coulibaly et al., 2009; Mand et al., 2009; Wanji et al., 2009; Johnston et al., 2014; Aljayyoussi et al., 2017). Therefore, anti-*Wolbachia* therapy is a promising avenue for the treatment and eradication of filariasis (Clare et al., 2019; Taylor et al., 2019; Johnston et al., 2021). The *Wolbachia* reside in the lateral cords in both sexes of adult worms but are also found in the ovaries and embryos of adult females (Kozek, 1977; McGarry et al., 2004; Landmann et al., 2010). The bacteria are required for the development, reproduction, and long-term survival of adult worms. It is likely that *Wolbachia* provide necessary metabolites from biological pathways that are incomplete or absent in the nematode genomes. These may include heme, riboflavin, nucleotide synthesis, and additional ATP for the host (Foster et al., 2005; Wu et al., 2009; Darby et al., 2012; Li and Carlow, 2012; Luck et al., 2016; Grote et al., 2017). In return, the worms may provide essential amino acids to the bacteria.

Sequences from *Wolbachia* and other bacterial symbionts have been found in eukaryotic host genomes, as a result of lateral gene transfer (LGT) (Hotopp et al., 2007; Ioannidis et al., 2013; Sieber et al., 2017). Nuclear *Wolbachia* transfers (*nuwts*) are found in over 80% of insect and nematode species infected with the bacteria. In *B. malayi*, there are hundreds of these *nuwts*, with over 10.6% of the *w*Bm genome integrated into the host nuclear genome. While many bacterial LGT sequences have deteriorated in host genomes, some of these transfers appear to be functional with actively transcribed protein coding genes across a wide range of eukaryotic species (Gladyshev et al., 2008; Acuña et al., 2012; Husnik et al., 2013; Ioannidis et al., 2013; Sieber et al., 2017).

To study the complex co-evolution between eukaryotes and their symbionts, it is necessary to obtain genome sequences from both species. In filarial endosymbionts, these genomes can be used to identify new drug targets. In other symbionts, the genomes can be used to identify novel molecular pathways related to their persistent infections and biological outcomes in eukaryotic hosts. Yet sequencing the genomes of intracellular bacteria is difficult because many cannot be cultured outside of the host or eukaryotic cell lines due to genome reduction (Wade, 2002; Fenollar et al., 2003; Foster et al., 2005; McCutcheon, 2010; Almeida et al., 2019). Even for bacteria with a high multiplicity of infection, the larger host genome results in substantial host DNA contamination and low relative levels of bacterial sequences. Field and clinical specimens can be limited, making it difficult to use standard sequencing methods with low sample input. Deep metagenomic sequencing can be used to assemble symbiont genomes, however this can be expensive and still may not result in sufficient bacterial sequences for *de novo* assembly. *Nuwts* and LGTs from the bacterial genomes can also be difficult to assemble if the sequencing depth is similar between species in a metagenomic sample. Techniques have been developed to enrich for bacterial DNA, particularly with *Wolbachia*, including pulsed-field gel purification (Sun et al., 2001; Foster et al., 2004; Wu et al., 2004; Foster et al., 2005), fluorescence-activated cell sorting (Thompson et al., 2013; Dam et al., 2020) and oligonucleotide probe hybridization (Kent et al., 2011; Melnikov et al., 2011; Geniez et al., 2012; Lefoulon et al., 2019). Unfortunately, these protocols are time consuming, require specialized equipment, require *a priori* knowledge of antibodies or sequences for probes, require expensive reagents, and/or require large quantities of input material.

Here, we present a novel application of ATAC-seq (Assay for Transposase-Accessible Chromatin using sequencing) for bacterial sequence enrichment to facilitate *de novo* genome assembly. Typically, ATAC-seq uses the Tn5 transposase to selectively cut and ligate sequencing adapters to accessible eukaryotic chromatin regions free of histones (Buenrostro et al., 2013). This leads to the enrichment of those “open” regions during sequencing, as the remaining majority of the genome is inaccessible to the transposase. Mitochondria and bacteria do not contain histones resulting in uniform Tn5 cutting across their genome. As a proof of principle, we performed ATAC-seq on *B. malayi* worms and show *de novo* genome assembly of the *Wolbachia* (*w*Bm) endosymbiont. Similar enrichment of bacterial sequences in published ATAC-seq datasets in human cells infected with *Mycobacterium M.tb* (Pacis et al., 2015) and *C. elegans* with *E. coli* strain OP50 contamination (Daugherty et al., 2017) enabled *de novo* assemblies of these bacterial genomes. This bacterial enrichment method should improve species identification and *de novo* metagenome assembly for a variety of host-associated microbiota.

## 2 Materials and methods

### 2.1 Tissue collection

Live adult *B. malayi* females and males were shipped overnight from the NIH-NIAID Research Reagent Resource Center (FR3) at the University of Georgia (Michalski et al., 2011). Upon arrival, the worms were transferred to prewarmed 37°C RPMI 1640 containing 10% heat inactivated fetal bovine serum (Thermo Fisher Scientific), 2 mM L-glutamine, 5 g/L glucose, 100 ug/mL streptomycin, 100 U/mL penicillin and 250 ng/mL amphotericin (Millipore Sigma) and incubated overnight at 37°C with 5% CO_2_. The worms were sorted into groups of 3 by sex and rinsed two times in 1X PBS. All liquid was removed, and the worms were frozen in 1.5 mL LoBind tubes with liquid nitrogen. All samples were stored at −80°C.

### 2.2 Nuclei isolation and *Wolbachia* immunostaining

The presence of *Wolbachia* cells in nuclei isolations were assessed using immunostaining for the *Wolbachia* surface protein (WSP). Three frozen adult female worms were placed on ice for 5 min prior to Dounce homogenization in 0.5 mL chilled 1X homogenization buffer (320 mM sucrose), 0.1 mM EDTA, 0.1% NP40 substitute (Millipore Sigma), 5 mM CaCl_2_, 3 mM Mg (Ac)_2_, 10 mM Tris pH 7.8, 167 μM β- mercaptoethanol, 1X protease inhibitor cocktail (Millipore Sigma) in water. Lysates were filtered through 200 μm then 40 μm filters to remove large tissue fragments and cuticle fragments, while retaining released nuclei and bacterial cells. The nuclei were pelleted by centrifugation at 500 x g for 10 min. The pellet was resuspended in 4 mL nuclei extraction buffer (10 mM Hepes pH 7.4, 1.5 mM MgCl_2_, 10 mM KCl, 1X protease inhibitor cocktail and 0.2% NP40 substitute) (Neely and Bao, 2019). The samples were split into 1 mL aliquots and added to poly-L-ornithine-treated (Millipore Sigma) glass cover slips (Thermo Fisher Scientific) by centrifugation at 500 x g at 4°C in a 12 well cell culture plate. The coverslips with adhered nuclei were fixed using 10% formalin for 10 min. The coverslips were washed 3 times with 1X PBS for 10 min and then blocked for 30 min in blocking buffer (0.5% BSA in 1X PBS). The Anti-*Wolbachia* surface protein (WSP) antibody (BEI resources) was diluted 1:1000 in blocking buffer and placed on the coverslips overnight at 4°C. To remove excess antibody, the coverslips were washed 3 times in 1X PBS with 0.05% Tween-20 for 10 min. A goat anti-mouse IgG conjugated with Alexa Fluor 488 (Abcam) was used as secondary antibody to localize WSP. All subsequent steps were performed in the dark. The antibody was diluted 1:2000 in blocking buffer and placed on the coverslips for 1 h at room temperature. The nuclei were washed 3 times with 0.05% Tween-20 in 1X PBS for 5 min and then washed an additional 2 times in 1X PBS. Coverslips were placed upside down on to a glass microscope slide with a droplet of Prolong Gold Antifade Mount with DAPI (Thermo Fisher Scientific). The nuclei were imaged using a Zeiss LSM 880 confocal microscope. The channel images were pseudo colored and stacked using ImageJ (Fiji v2.1.0).

### 2.3 Nuclei isolation and ATAC-seq library preparation and sequencing

The Omni-ATAC-seq protocol (Corces et al., 2017) was adapted for fresh frozen nematodes. All steps were performed on ice unless noted otherwise. We prepared 2 biological replicates for each sex, with each replicate containing a pool of 3 worms. The frozen male and female worm samples were Dounce homogenized in 1X homogenization buffer (recipe in previous section) separately. The lysates were sequentially filtered through 70 μm, 40 μm and 20 μm filters, removing worm cuticles and undisrupted embryos. Nuclei were pelleted by spinning at 500 x g for 10 min in a fixed angle centrifuge at 4°C. After removing the supernatant, the pellet was resuspended in 1 mL ATAC Resuspension buffer (10 mM Tris–HCl pH 7.4, 10 mM NaCl, 3 mM MgCl_2_, 0.1% Tween-20) and pelleted again. The supernatant was completely removed, and the pellets were each resuspended in transposition mix containing 25 μL 2X tagmentation buffer (Diagenode), 2.5 μL loaded tagmentase (Diagenode), 16.5 μL 1X PBS, 0.5 μL 1% digitonin, 0.5 μL 10% Tween-20, and 5 μL ultrapure water. The samples were incubated at 37°C for 30 min at 1000 rpm in a thermomixer (Eppendorf). The tagmented DNA was cleaned up using the Monarch PCR and DNA Cleanup Kit (New England BioLabs) and eluted in 20 μL of ultrapure water. The libraries were indexed and amplified with 10 PCR cycles using unique dual indexes (Diagenode) and NEBNext High-Fidelity 2X PCR Master Mix (New England BioLabs). The libraries were size selected using consecutive AMPure XP bead (Beckman Coulter) clean ups by removing fragments bound to 0.5X beads and then keeping fragments bound to 1.0X beads. The samples were pooled and sequenced on a single flow cell of the Illumina NextSeq 550 to a depth of at least 20 million reads per library.

### 2.4 Genomic DNA library preparation and sequencing

Two biological replicates of genomic DNA (gDNA) from 10 adult female *B. malayi* worms were extracted using the Monarch High Molecular Weight DNA Extraction Kit for Tissue (New England BioLabs) using the standard protocol. DNA was run on a Pippin Pulse gel (Sage Science) and had an average length of 100 kb. The genomic DNA libraries were made following the Nextera DNA Library Prep Kit protocol (Illumina), starting with 50 ng of DNA. Replicate 1 was tagmented for 5 min and replicate 2 was tagmented for 15 min with 5 μL TDE1 at 55°C in the thermomixer. The reaction was cleaned using the Monarch PCR and DNA Cleanup kit. The libraries were indexed and amplified using Nextera dual indexes (Illumina) and NEBNext High-Fidelity 2X PCR Master Mix with 10 PCR cycles. Excess adapters and primers were removed by cleaning up with 1.5X AMPure XP beads (Beckman Coulter). The samples were pooled at equal concentrations and sequenced on a single flow cell of the Illumina NextSeq 550 to a depth of at least 20 million reads per library.

### 2.5 ATAC-seq analyses

Default options for each analysis tool were used unless noted otherwise. The paired-end reads from all libraries were trimmed to remove remaining adapter sequences and low quality bases using Cutadapt (v1.16) (Martin, 2011) with the –paired and –nextera options. The read quality was assessed using FastQC (v0.11.9) (Andrews, 2023). Some reads may map with equal quality to both the *w*Bm and *B. malayi* genomes as a result of *nuwt* sequences. Therefore, we aligned all of the trimmed reads to both the reference *B. malayi* chromosomes (GCF_000002995.4) and the *w*Bm genome (AE017321.1) separately and calculated the mapping percentages using Bowtie2 (version 2.4.5) (Langmead and Salzberg, 2012). To identify which reads map to both genomes, we first used “Samtools view -F 4 | cut -f 1” (v1.15.1) (Li et al., 2009) to extract mapped read names from each of the BAM files. The Unix “comm” command was then used to find the read names shared between the *w*Bm and *B. malayi* mapped BAM files for each library. The proportion of shared reads was calculated by counting the number of common read names and dividing that by the total number of reads in each library. We retained the shared mapped reads in their respective BAM files for further analyses. Picard (v2.27.5) (Broadinstitute/picard Broad institute, 2023) was used to calculate the insert size (CollectInsertSzeMetrics) and to mark PCR duplicates (MarkDuplicates) in the BAM files. We used Deeptools (v3.5.1) (Ramírez et al., 2014) to make BigWig files (BamCoverage) of the ATAC-seq and gDNA libraries for visualization in IGV (v2.11.9) (Thorvaldsdóttir et al., 2013) and to calculate the sequencing depth (plotCoverage) for reads aligned to the *B. malayi* nuclear and *Wolbachia* chromosomes. We called peaks in the ATAC-seq samples aligned to the *B. malayi* nuclear chromosomes using MACS2 (v2.2.7.1) (Zhang et al., 2008) with an FDR cutoff for 0.05. ATAC-seq peaks that overlap with *nuwt* regions in the *B. malayi* genome were removed using Bedtools intersect (v2.30.0) (Quinlan and Hall, 2010), as we cannot determine if the reads originate from *B. malayi* or *Wolbachia* DNA. The BAM and filtered peak files were loaded into R using Diffbind (v3.4.11) (Stark and Brown, 2023) to create a read count matrix across all ATAC-seq samples for each peak region. This count matrix in Diffbind was then used to calculate the FRiP (fraction of reads in peaks) and Pearson correlation between replicates. We used ChIPseeker (v1.30.3) (Yu et al., 2015) to annotate ATAC-seq peaks to genomic features, with promoters calculated as 1 kb up and downstream of the first base in the gene model.

### 2.6 *Wolbachia de novo* genome assembly and assessment

Trimmed reads for female ATAC-seq and gDNA libraries were randomly downsampled to 10 million, 1 million, 500 thousand and 100 thousand total reads using Seqtk subseq (v1.3) (Li, 2023). We used SPAdes (v3.15.4) (Nurk et al., 2017; Prjibelski et al., 2020) to make metagenomic assemblies from the downsampled libraries with the “meta” setting. We mapped the reads from each library back to its respective assembly to obtain sequencing depth for each contig. Each assembly was aligned to the NCBI Nucleotide (NT) database (v5) containing *Wolbachia* genomes and to the *B. malayi* genome (GCF_000002995.4) using BLASTN (v2.13.0) (Altschul et al., 1990; Camacho et al., 2009). Blobtoolkit (v3.2.7) (Laetsch and Blaxter, 2017; Challis et al., 2020) was used to visualize and bin the ATAC-seq and gDNA assemblies (with 10 million input reads) by BLAST results, sequence coverage and GC content. Using the Blobtoolkit viewer, we filtered the gDNA and ATAC-seq assemblies using multiple metrics. First, all contigs with a length less than 500 bp were removed, as these are difficult to bin correctly (Strous et al., 2012; Gurevich et al., 2013; Vosloo et al., 2021). For the gDNA assembly, the contigs that aligned to bacterial sequences with BLASTN were kept to bin *Wolbachia* contigs. With the ATAC-seq assembly, all contigs with over 1,000X coverage were kept to isolate *Wolbachia* contigs from nematode sequences. The few remaining *B. malayi* (rRNA regions) and mitochondrial contigs were also removed based on BLAST results. Quast (v5.2.0) (Gurevich et al., 2013) was used to assess the genome qualities, with the published *w*Bm genome (AE017321.1) and annotation used as a reference (Foster et al., 2005). With Quast, we identified the percentage of the genome assembled, misassembles, mismatches, N50, genome features, and contig number. BUSCO (v5.4.2) (Simão et al., 2015; Manni et al., 2021) with the bacteria odb10 database was also used within QUAST to assess genome completeness, comparing the gene content between the *w*Bm reference and our new assemblies. We then used D-genies (v1.5.0) (Cabanettes and Klopp, 2018; Li, 2018) with Minimap2 alignment to visualize the alignment of our binned assemblies and the reference genome. BLASTN was used to map the binned assemblies to the reference *w*Bm genome using output format 6. Columns 1, 7 and 8 were kept, creating a bed file to view the coordinates of gaps in our new assemblies using IGV. GenMap (v1.3.0) (Pockrandt et al., 2020) was used to identify repeats in the reference genome, by calculating the mappability of each region using 75 bp k-mers, the same length as our sequencing reads. Using Bedtools intersect (v2.30.0) (Quinlan and Hall, 2010), we found overlaps between the gaps in our new assemblies and the repeat regions of the reference genome. Bedtools intersect was also used to calculate overlap between mismatches and SNPs (identified with QUAST) with *nuwts* in the *w*Bm genome. The repeat length, score and assembly status were visualized using ggplot2 (Valero-Mora, 2010).

### 2.7 Genome assembly with published ATAC-seq data

SRA-toolkit (v2.11.1) was used to download ATAC-seq runs from *C. elegans* (SRR5000677) (Daugherty et al., 2017) and human dendritic cells infected with *M. tb* (SRR1725731) (Pacis et al., 2015). The raw reads were trimmed with Cutadapt as described above (Section 2.5). We used Kraken2 (v2.1.3) (Wood et al., 2019) to classify the trimmed reads by taxonomy. The reads were then assembled with Spades as described above (Section 2.6). The trimmed reads were mapped back to the new assemblies using Bowtie2 to calculate sequencing depth. The assemblies were aligned to the NCBI NT database using BLASTN. The assemblies, coverage files and BLASTN results were visualized with Blobtoolkit.

## 3 Results

### 3.1 Prescence of *Wolbachia* cells after nuclei isolation

**Figure 1:**
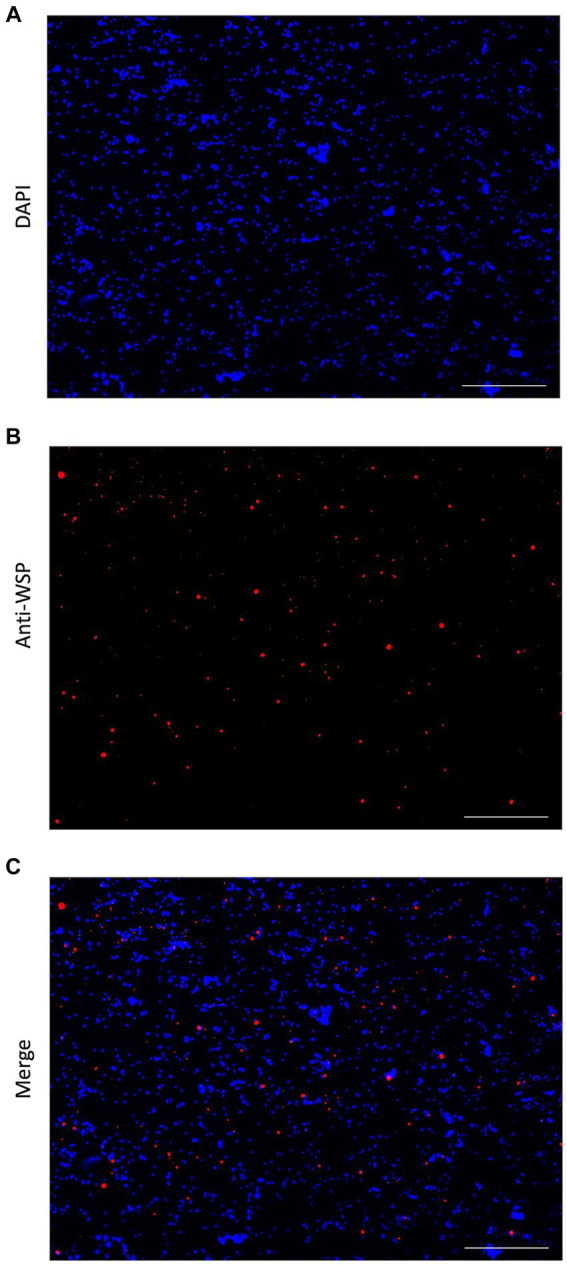
Immunofluorescent detection of *Wolbachia*. **(A)** DAPI stain (blue) of isolated *Brugia malayi* nuclei. **(B)** Anti-WSP detection in the nuclei isolation using an anti-mouse IgG secondary antibody conjugated with Alexa Fluor 488 (pseudo colored red). **(C)** Merged image, showing the presence of *Wolbachia* cells in *Brugia malayi* nuclei extraction. All images at 40X, Scale = 100 μm.

ATAC-sequencing using the Tn5 transposase was performed on adult *B. malayi*, containing *Wolbachia* endosymbionts. Nuclei isolation is the first step in ATAC-seq library preparation. *Wolbachia* presence after cell membrane lysis was confirmed with immunofluorescent staining. *Wolbachia* cells were stained using the anti-WSP antibody, while *B. malayi* nuclei were stained with DAPI. Intact *Wolbachia* cells can be seen amongst the nematode nuclei, confirming that *Wolbachia* DNA will be present during ATAC-sequencing (Figure 1).

### 3.2 *Brugia malayi* chromatin accessibility

**Figure 2:**
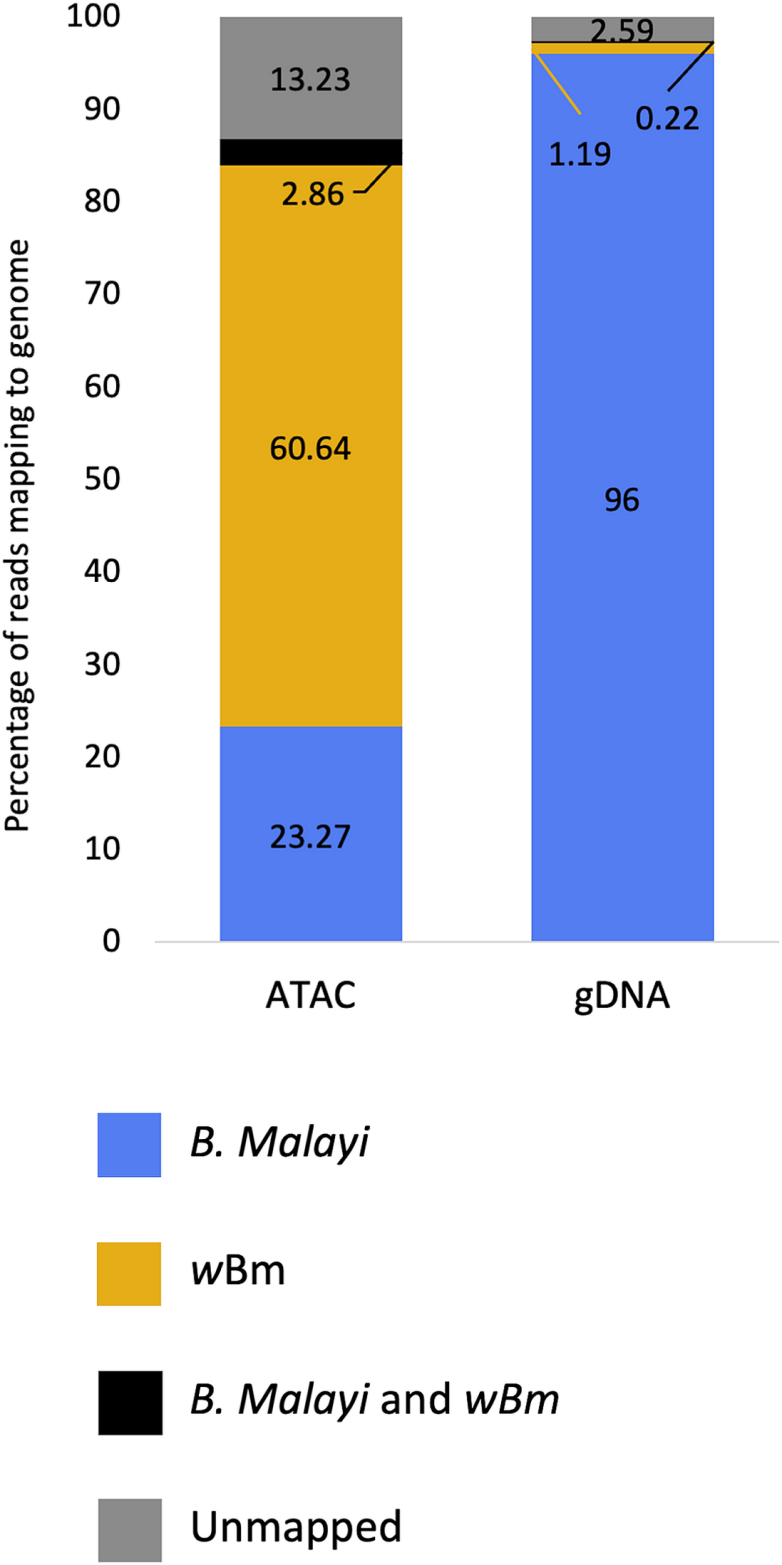
Mapping statistics of ATAC-seq and gDNA libraries from *Brugia malayi* samples containing *Wolbachia*. The proportion of reads mapping to the *Brugia malayi* and mitochondrial chromosomes only is shown blue. The proportion of reads mapping to the *Wolbachia* genome only is shown in yellow. The proportion of reads that multi-map to both the *Brugia malayi* and *w*Bm chromosomes are shown in black. Reads that are unmapped are shown in grey.

ATAC-seq and gDNA libraries were aligned to both the *B. malayi* and *Wolbachia* genomes. Both libraries had an average insert size of 100 bp (Supplementary Figure S1). The Tn5 transposase cuts and attaches sequencing adapters to DNA that is not surrounded by histones. Since bacterial genomes do not contain histones, we expected an enrichment of *Wolbachia* reads in ATAC-seq libraries. We found that at least 60% of reads mapped to the *Wolbachia* genome in the adult female ATAC-seq libraries, while only 1.19% of reads were from the bacteria in the standard gDNA libraries (Figure 2; Supplementary Table S1). This represents an over 50-fold enrichment of bacterial reads when transposase library preparation is used on samples containing intact chromatin. 23.27% of reads map only to the *B. malayi* nuclear and mitochondrial chromosomes in the ATAC-seq library, with 96% of reads mapping to these chromosomes in the gDNA library. A small proportion of reads map to both the *B. malayi* nuclear and *w*Bm chromosomes. These most likely represent reads mapping to *nuwts*, as these sequences are found in both species. There is a higher number of these dual-mapped reads in the ATAC-seq library. The bulk of these reads are likely originating from *Wolbachia*, as a result of the overall enrichment of the bacterial sequences. The remaining reads are unassigned or map to the gerbil genome, which is the laboratory host for the nematode.

**Figure 3:**
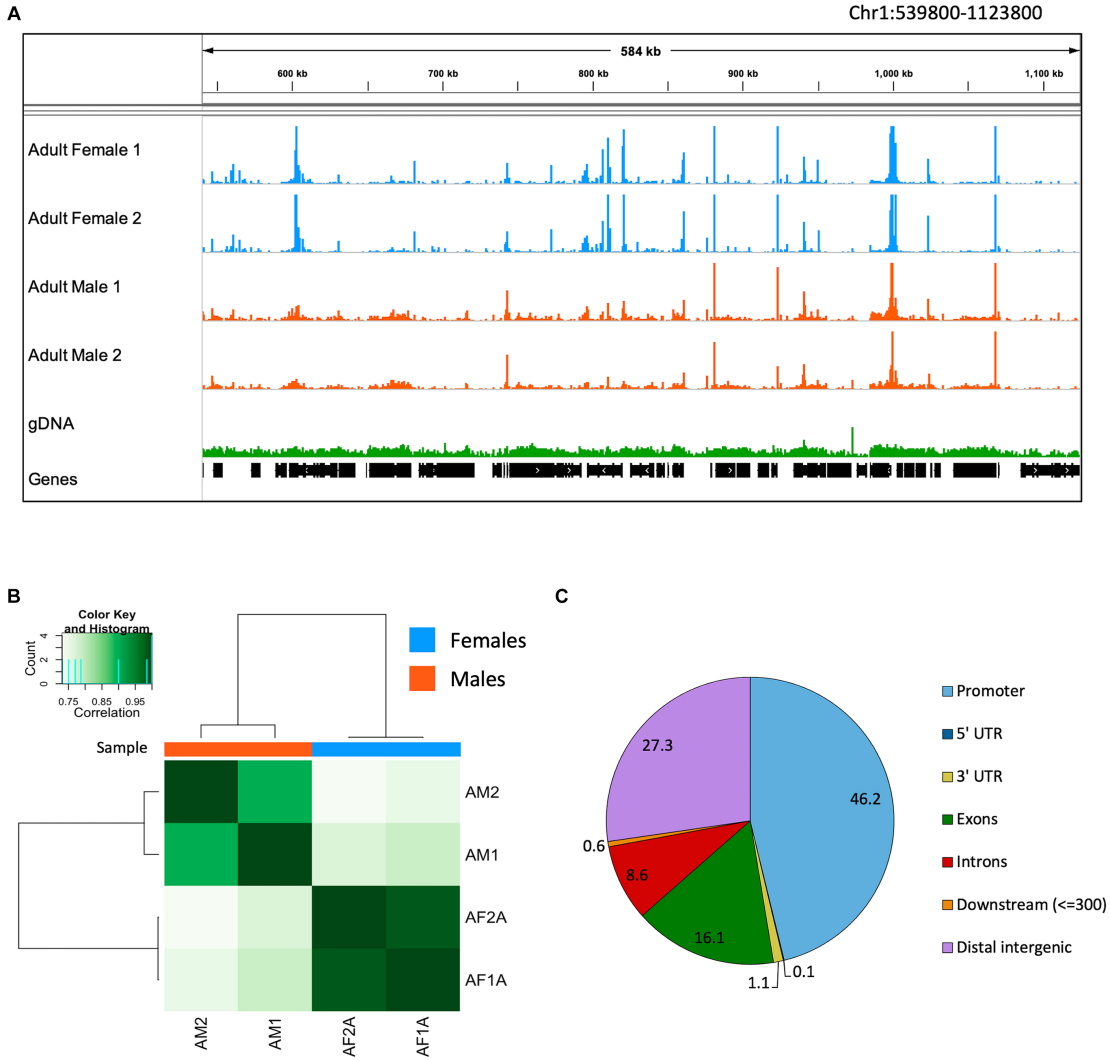
*Brugia malayi* chromatin accessibility. **(A)** IGV trace showing an example of ATAC-seq and gDNA read alignments in *Brugia malayi* Chr1. Adult female ATAC-seq replicates are shown in blue, adult males in orange and gDNA in green. Genes are shown in black. The scale for the ATAC-seq libraries is 0–1,500 and 0–500 for gDNA. **(B)** Heatmap showing the Pearson correlation between ATAC-seq samples. Dark colors correspond to higher correlation coefficients. **(C)** Pie chart showing proportion of ATAC-seq peaks mapped to each genomic feature.

When mapped on the *B. malayi* nuclear genome, ATAC-seq reads pile up in distinct regions called peaks, while gDNA reads map relatively uniformly across the chromosomes (Figure 3A). The ATAC-seq peaks correspond to open chromatin regions where transcription factors can bind the DNA and actively regulate gene expression. Many peaks are shared across males and females. However, some peaks can only be found in one sample type, resulting in a unique chromatin landscape dependent on the nematode sex. The read coverage distribution was calculated for ATAC-seq and gDNA reads mapped to the *B. malayi* chromosomes (Supplementary Figure S2). The gDNA libraries have a bell-shaped coverage distribution curve, with an average depth of 25 reads. Both the male and female ATAC-seq libraries have an L shaped coverage distribution curve with an average depth of 16.8 and 9.7 reads, respectively. This means that the gDNA libraries have a more uniform coverage across all of the *B. malayi* chromosomes, while most genomic regions have little to no coverage in the ATAC-seq samples, as expected.

Using Pearson correlation, we find that biological ATAC-seq replicates cluster together, showing the reproducibility of our data with this optimized ATAC-seq method (Figure 3B). The clustering also shows that males and females have global differences in the chromatin accessibility. The ATAC-seq peaks were assigned to genomic features using the *B. malayi* genome annotation (Figure 3C). Promoter regions (defined as 1 kb up- and downstream of the first base in the gene model) had the greatest number of peaks at 46.2%. Distal intergenic regions, which generally correspond to enhancers, have 27.3% of the peaks. The other 26.5% of peaks fall in genic regions, including untranslated regions, exons, and introns. An enrichment of peaks at promoter regions has been observed in other ATAC-seq datasets. In mammals, around 10–25% of peaks are found in promoter regions (Chung et al., 2018; Yan et al., 2020). *Drosophila melanogaster* data is more similar to *B. malayi*, where 40–50% of peaks are found in promoters (Santiago et al., 2021; Dhall et al., 2023).

### 3.3 Enrichment of *Wolbachia* reads in *Brugia malayi* ATAC-seq

**Figure 4:**
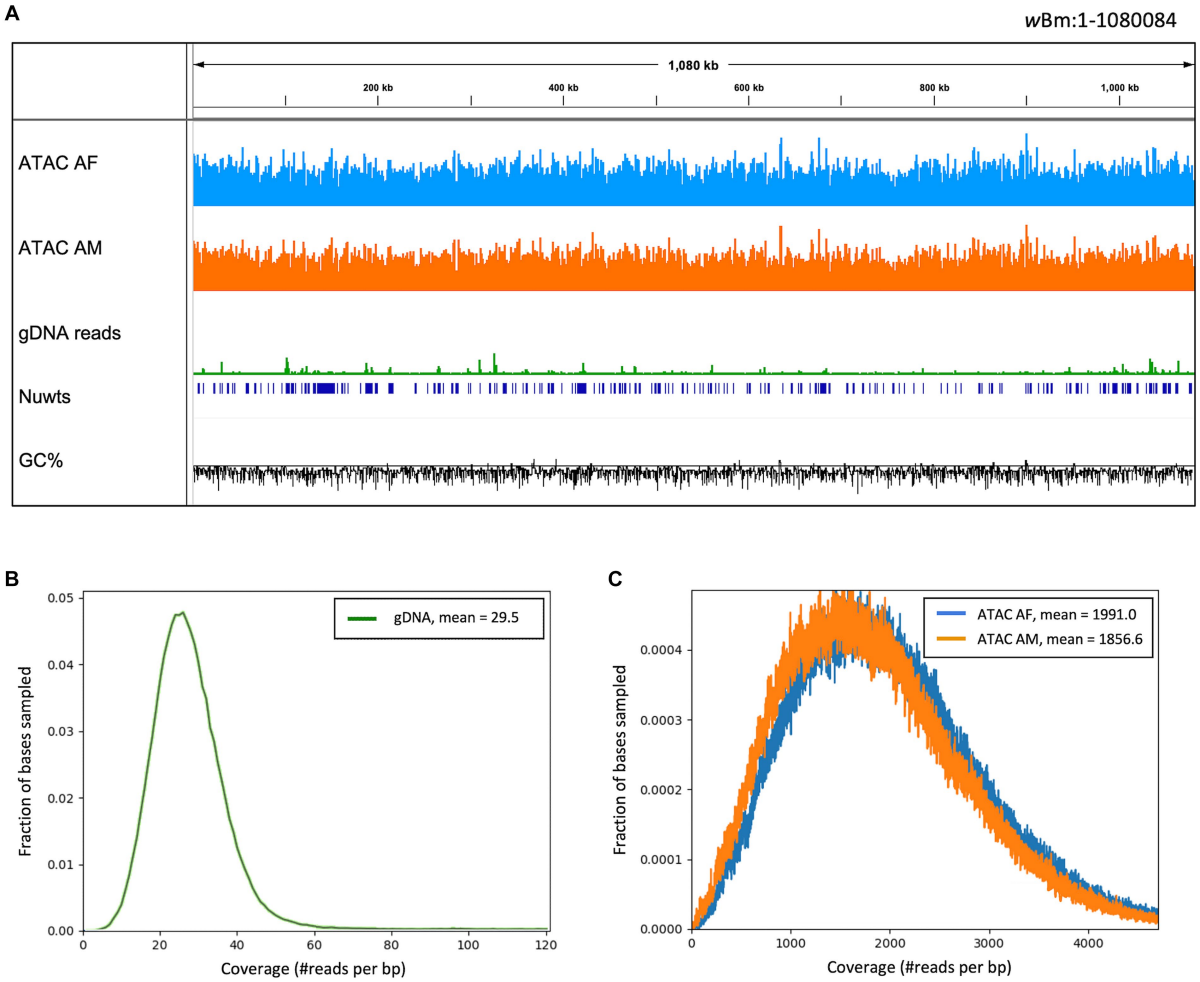
Read coverage across the *Wolbachia* genome. **(A)** IGV trace showing read alignment across the *w*Bm genome. ATAC-seq library tracks have a scale of 0–7,500 reads and the gDNA track has a scale of 1,000 reads per 50 bp bin. The nuclear transfer origins are shown in dark blue. GC fraction is shown in black with a scale of 0–1 with the solid black line representing 0.5. **(B)** Distribution of gDNA read depth across the *w*Bm genome, with an average depth of 29.5. **(C)** Distribution of ATAC-seq read depth across the *w*Bm genome. The female library has an average depth of 1991 reads and the male library has an average depth of 1856.6 reads. Colors correspond to sample type with female ATAC-seq in blue, male ATAC-seq in orange and gDNA in green. 100,000 bases were randomly sampled across the *w*Bm reference genome for **(B,C)**.

As mentioned in the previous section, there was an enrichment of *Wolbachia* reads in the ATAC-seq libraries compared to the gDNA libraries. We found that the read coverage is almost uniform across the *Wolbachia* genome in all of the libraries, with much higher sequencing depth in the ATAC-seq libraries (Figure 4A). The average coverage distribution across the *Wolbachia* genome was calculated for all libraries (Figures 4B,C). The gDNA libraries had a bell-shaped distribution, with an average depth of 29.5 reads, similar to that observed for the nematode chromosomes (Figure 4B; Supplementary Figure S2). The male and female ATAC-seq libraries also had a bell-shaped distribution, with an average read depth of 1856.6 and 1991 reads, respectively (Figure 4C). The sequencing depth and coverage from the ATAC-seq libraries is much higher in the *Wolbachia* chromosome than in the *B. malayi* chromosomes (Supplementary Figure S2). There are some regions with higher read depth in the *w*Bm genome with both library preparation methods, however, these regions have different coordinates in the ATAC-seq and gDNA libraries. There is a slight GC (guanine + cytosine) bias in the ATAC-seq libraries, leading to higher read coverage in genomic regions with higher GC content (Supplementary Figure S3). The gDNA libraries have an increase in sequencing depth over *nuwts*, with almost twice as many average reads compared to regions that have not been transferred to the *B. malayi* nuclear genome (Supplementary Table S2). ATAC-seq libraries have uniform depth between *nuwts* and the rest of the *w*Bm genome. Therefore, reads from *B. malayi nuwts* artificially increase read depth in gDNA libraries, while they do not appear to have any effects on ATAC-seq mapping. This means that ATAC-seq reads mapping to the *nuwts* in the *w*Bm genome most likely originate from *Wolbachia*. There is a similar distribution of reads mapping to the *w*Bm and *B. malayi* chromosomes in the adult male and adult female ATAC-seq samples (Supplementary Table S1). While the larger females contain an overall higher number of *Wolbachia*, the ratio of *Wolbachia* cells to nematode nuclei appear to be similar between the two sexes (McGarry et al., 2004). Additionally, the *Wolbachia* containing embryos were removed during nuclei isolation, resulting in a loss of the embryonic *Wolbachia* DNA in the female libraries. Therefore, it is unsurprising that the adult female samples do not have a significantly higher proportion of *Wolbachia* reads than the adult males.

### 3.4 *Wolbachia de novo* genome assembly

**Figure 5:**
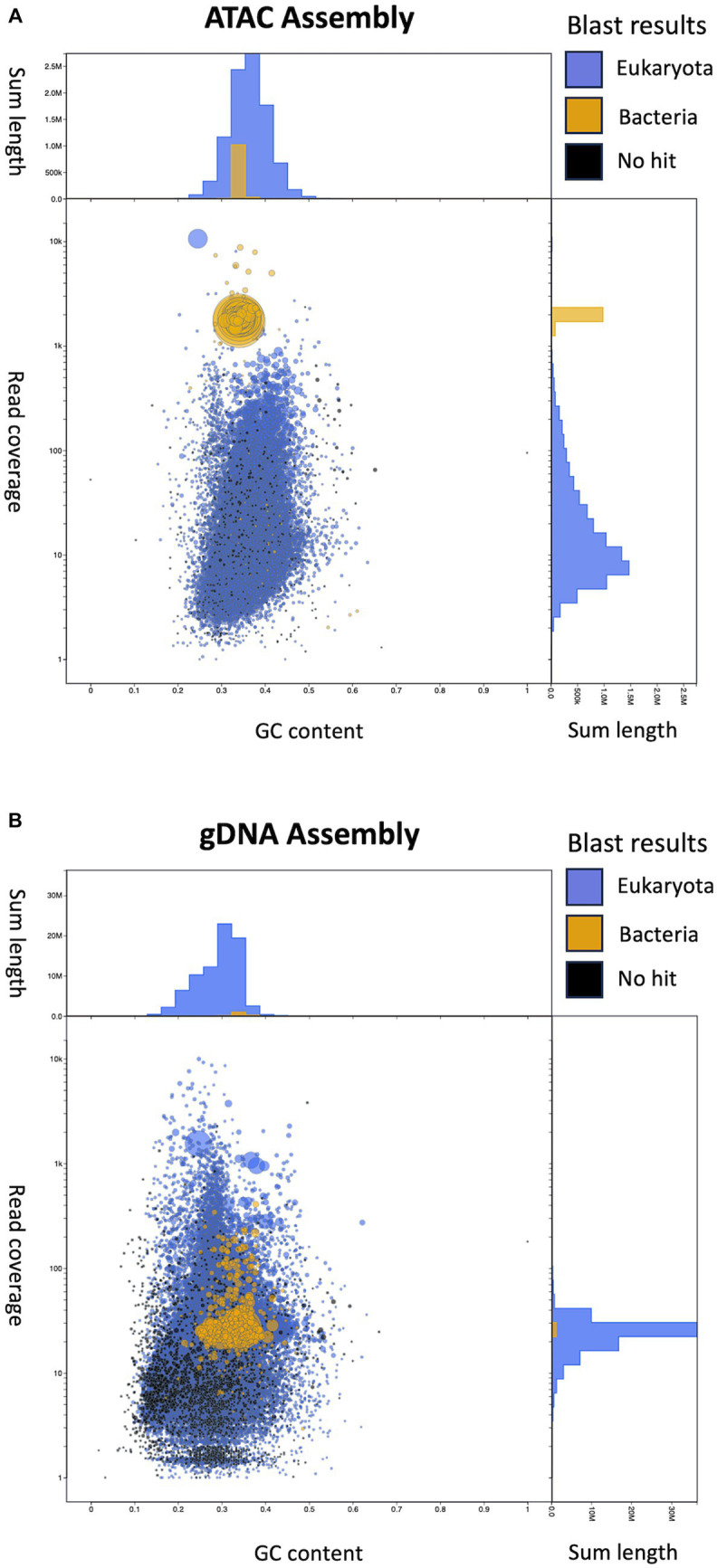
Visualization of assemblies using Blobtools. **(A)** Blobtools plot showing Female ATAC-seq *de novo* assembly. **(B)** Blobtools plot showing gDNA *de novo* assembly. In both plots, contigs are represented by circles, where the size corresponds to the length of the contig. The color of the circles displays the BLAST results, where blue contigs match eukaryotic sequences, yellow contigs match bacterial sequences and black contigs do not match to any sequences in the NCBI Nucleotide database. The *x*-axis is the GC content of the contigs. The *y*-axis is the coverage of the contigs, after the starting reads are mapped back onto the new assemblies. The *y*-axis labels are the actual values, while the distance between tick marks is on a log_10_ scale. The length of the contigs is summed by GC content bins (top of each plot) and by converge bins (right of each plot).

Metagenomic assemblies for female ATAC-seq and gDNA libraries were created using metaSpades(Nurk et al., 2017) from 10 million randomly subsampled reads. We used Blobtools to visualize and bin our assemblies based on GC content, BLASTN alignment, and sequencing depth (Figure 5) (Laetsch and Blaxter, 2017; Challis et al., 2020). The plots show each contig represented as a circle, where the size represents the length of the contig, and the color represents the BLAST results. GC content and read coverage are represented on the x and y-axis, respectively. In both the ATAC-seq and gDNA assemblies, the contigs cannot be binned by GC content, as both the *B. malayi* and *Wolbachia* genomes are AT rich with similar GC percentages (25 and 34%, respectively) (Foster et al., 2005; Tracey et al., 2020). In the ATAC-seq assembly, the contigs can be binned by both coverage and BLAST results alone (Figure 5A). A combination of the two can be used to remove the few remaining *B. malayi* contigs from the coverage binning method. The contigs assigned to *B. malayi* with high coverage contain the ribosomal RNA (rRNA) tandem repeat, which appears to be completely open in *B. malayi* chr2 (Supplementary Figure S4), and the complete mitochondrial chromosome, which located outside of the nucleus and does not contain histones (Supplementary Figure S4). The gDNA assembly cannot be binned using coverage, as there is uniform coverage throughout both *B. malayi* and *Wolbachia* chromosomes (Figure 5B). Therefore, the contigs from the ATAC-seq assembly were binned using a coverage cutoff of 1,000, with the *B. malayi* mitochondria and rRNA tandem repeats removed, while the contigs from the gDNA assembly were binned by BLAST results only.

**Figure 6:**
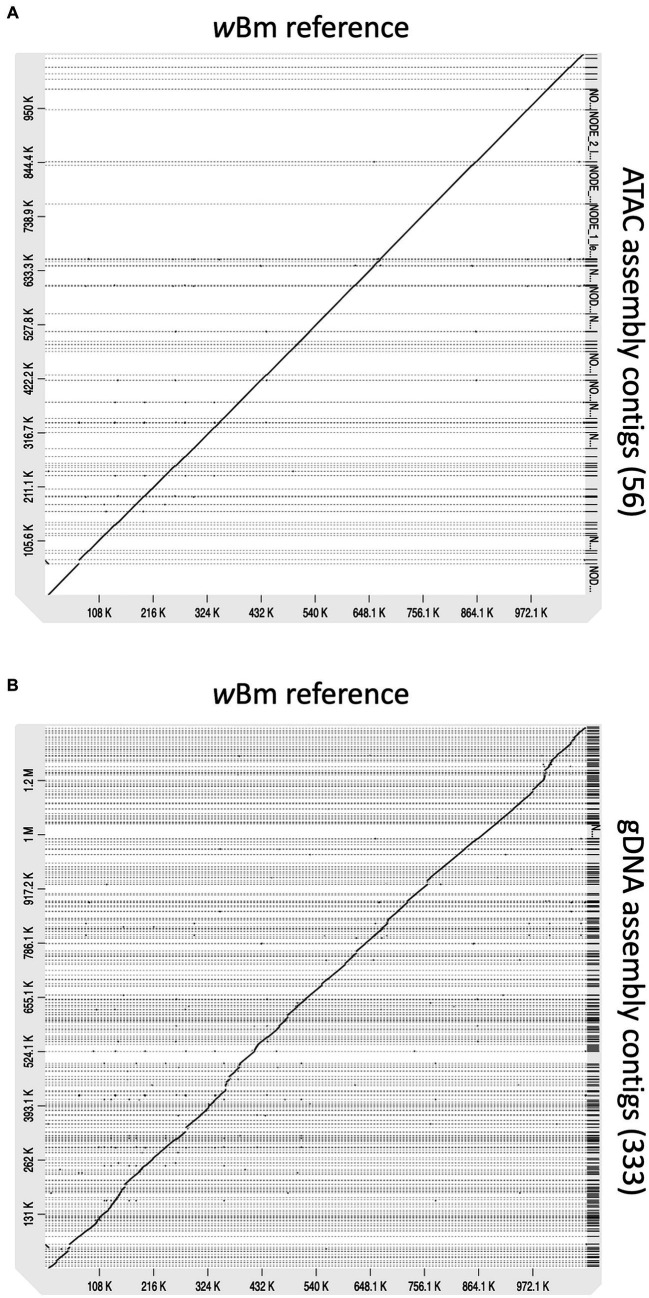
Alignments of new assemblies to the *w*Bm reference genome. **(A)** ATAC-seq assembly with 56 contigs mapping to the *w*Bm reference genome. **(B)** gDNA assembly with 333 contigs mapping to the *w*Bm reference genome. The genomes were aligned using minimap2 and the dot plots were created with D-genies. All contigs are sorted based on the coordinates of the reference genome. Grey dashed lines represent contig boundaries.

We end up with a more complete and correct assembly using ATAC-seq libraries compared to the gDNA library (Table 1; Figure 6). The ATAC-seq assembly has 56 contigs that align to the *w*Bm genome, covering over 97% of the reference (Figure 6A). The gDNA assembly still covers 96% of the *w*Bm genome, however the assembly is much more fragmented with 333 contigs aligning to the reference (Figure 6B). The ATAC-seq assembly has larger contigs, resulting in a much higher N50 value compared to the gDNA assembly, with 37,139 bp versus 7,533 bp, respectively (Table 1). The gDNA library has 9 misassemblies, including inversions, relocations, and translocations, while the ATAC-seq assembly has none. Additionally, the DNA from both the gDNA and ATAC-seq libraries were derived from the same batch of worms yet the gDNA library has 308 nucleotides that do not match the *w*Bm reference genome and the ATAC-seq library only has one nucleotide change. 87.4% of the nucleotide mismatches in the gDNA assembly fall within *nuwts* and are most likely the result of nucleotide changes in the host nuclear genome. The ATAC-seq mismatch falls outside of *nuwts* and is either a real nucleotide change or is the result of a PCR error.

**Table 1:** Genome quality statistics for the new assemblies calculated using QUAST.

Assembly	Genome fraction	# of contigs	Largest contig	N50	Misassemblies	Mismatches
ATAC-seq	97.4%	56	106,937	37,139	0	1
gDNA	96.0%	333	35,983	7,533	9	308

Although we had an average sequencing depth of almost 2000X, there are still some gaps present in the ATAC-seq assembly. To determine whether the gaps are from repetitive regions or low read coverage, we first identified repeats in the *w*Bm reference genome. These regions may be more difficult to assemble with short read sequencing. Repeat scores of the genomic sequences were calculated by taking the inverse of the mappability score (1 – m, where m = mappability). We mapped our ATAC-seq contigs onto the reference genome to identify the coordinates of our gaps and compared this to the sequencing coverage and repetitive regions (Supplementary Figure S5A). The gaps overlap with the repeats and are not correlated with read coverage and depth. However, not all repeats resulted in gaps in the assembly. When the repeats were plotted by repeat score and repeat length, we found that repeats longer than 163 bps were not assembled, except for two repeats with lengths of 557 and 584 bps (Supplementary Figure S5B). Therefore, gaps are generally caused by repeats significantly longer than our average fragment size and are not a result of low read coverage.

### 3.5 *De novo* genome assembly with decreasing starting sequencing reads

Deep sequencing of samples containing endosymbionts has been previously used to assemble unculturable bacterial genomes (Kumar and Blaxter, 2011; Mackelprang et al., 2011). These datasets contain hundreds of millions of reads, resulting in high sequencing and computational costs. We were able to obtain a high-quality assembly using only 10 million reads from our ATAC-seq dataset. Here, we further subsampled the starting read numbers to determine how many reads are necessary for *Wolbachia* genome assembly using both ATAC-seq and standard gDNA methods. The original ATAC-seq and gDNA libraries were randomly downsampled to 20, 10, 1, 0.5, 0.25 and 0.1 million total reads. These libraries with decreasing read numbers were then assembled using metaSpades (Nurk et al., 2017), as described in the previous section.

**Figure 7:**
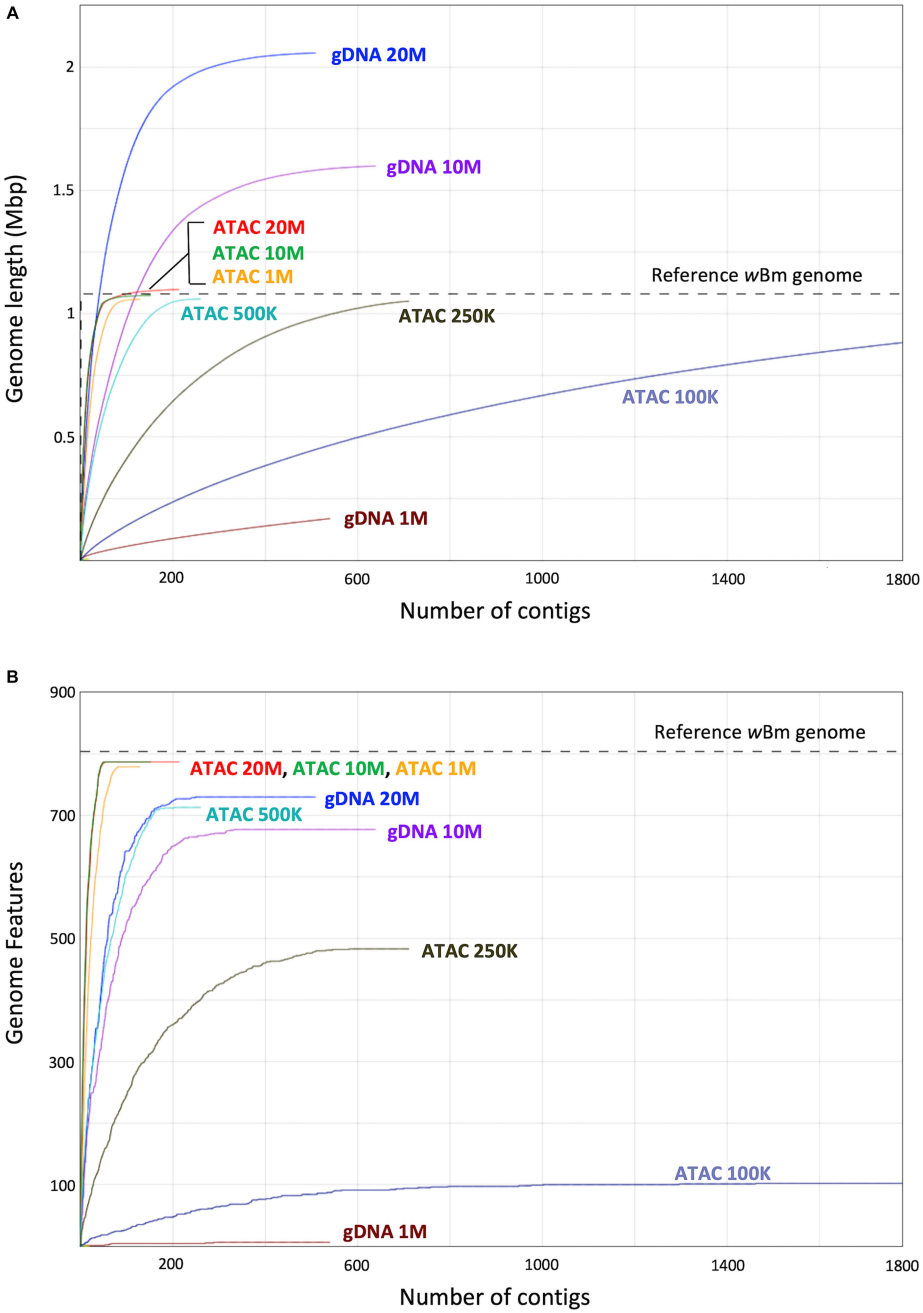
Genome length and genomic feature calculations from assemblies with decreasing input reads. **(A)** Graph showing the length of assemblies with decreasing starting reads compared to the *w*Bm reference genome. The *x*-axis represents the number of contigs, and the y-axis represents the sum length of the contigs in millions of base pairs. **(B)** Graph showing the number of genomic features (genes) found in each assembly compared to that of the *w*Bm reference genome. The *x*-axis represents the number of contigs, and the *y*-axis represents the sum of the genomic features assembled. The color of the line represents the assembly. In both panels the grey dotted line corresponds to the corresponding value in the *w*Bm reference genome.

The metagenomic assemblies were evaluated and compared to the *w*Bm reference genome using QUAST (Gurevich et al., 2013). We determined the length of each new assembly and compared it to that of the reference genome (Figure 7A). The gDNA assemblies created with 20 and 10 million starting reads resulted in an over assembly of the *Wolbachia* genome, indicating sequences are either inappropriately duplicated or *B. malayi* sequences are incorrectly added into the bacterial assembly because of *nuwts*. The gDNA assembly created with 1 million reads is much shorter than the reference, meaning there is not enough sequencing coverage to assemble the bacterial genome. The ATAC-seq genomes made with 20, 10, 1 and 0.5 million reads are all assembled to a similar length as the reference. The ATAC-seq data does not have the issue with over assembly when higher numbers of starting reads are used, likey because of increased correct reads originating from *Wolbachia* cells and little to no reads from the *B. malayi nuwts*. Even at 250 thousand reads, the ATAC-seq assembly results in an almost complete genome but with a larger number of contigs.

Another important aspect of genome quality is the presence of genomic features, such as genes. We identified the number of genomic features found in our new assemblies and compared these values to the *w*Bm reference annotation which has 804 genomic features (Foster et al., 2005) (Figure 7B). The ATAC-seq assemblies with 10 and 20 million reads both have 787 genomic features assembled, while the genome from 1 million reads has slightly fewer with 779. The gDNA assembly with 20 million reads has only 730 genomic features, despite having a longer genome length. The genes present in the gDNA assemblies drop down to 677 with 10 million reads and only 6 genes assembled with 1 million starting reads. Therefore, the use of ATAC-seq for bacterial symbiont assembly results in higher quality genomes and requires a lower number of reads, resulting in less expensive sequencing costs.

### 3.6 Bacterial enrichment in published ATAC-seq datasets

**Figure 8:**
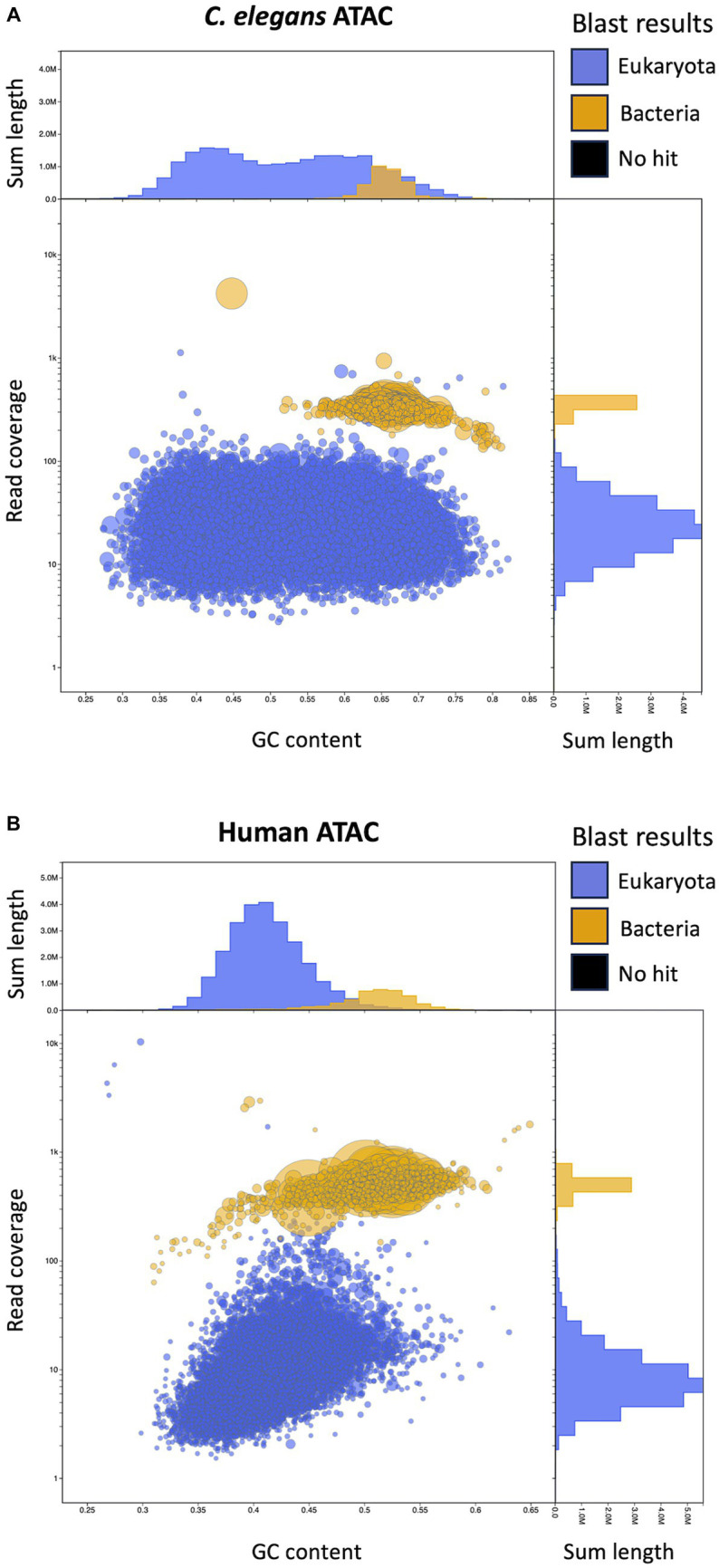
Visualization of *C. elegans* with *E. coli* and Human with *M. tb* ATAC-seq assemblies with Blobtools. **(A)** Blobtools plot showing *de novo* genome assembly from published *C. elegans* ATAC-seq data contaminated with OP50 *E. coli*. **(B)** Blobtools plot showing *de novo* genome assembly with published ATAC-seq data from human DCs infected with *M. tb*. Further details for the plot organization are described in the Figure 5 legend.

Having determined that ATAC-seq performs efficiently as a bacterial sequence enrichment method for *B. malayi* and *Wolbachia*, we wanted to determine if this method works across diverse bacteria and eukaryote pairs using published ATAC-seq datasets (Pacis et al., 2015; Daugherty et al., 2017) (Supplementary Figure S6). Daugherty et al., published an ATAC-seq dataset in *C. elegans* across life cycle stages (Daugherty et al., 2017). We focused on the young adult sample (SRR5000677), as the nematodes at this life cycle stage consume OP50 *E. coli* as their primary food source. The dataset had 82.8% of total reads originating from *E. coli* DNA, with only 16.5% of reads mapping to *C. elegans*. Pacis et al., identified open chromatin in human dendritic cells (DCs) infected with *M. tb* using ATAC-seq (Pacis et al., 2015). The study used 5 *M. tb* cells per individual human DC. When taking into account the length of their respective genomes and the 5:1 ratio of bacterial to eukaryotic cells, we would expect 0.6% of the reads to originate from bacterial DNA if no enrichment method is used. This dataset (SRR1725731) contains 31.8% bacterial reads and 67.4% human reads. This results in a 50-fold enrichment of bacteria sequences over the expected value, similar to the enrichment we report here for *B. malayi* and *Wolbachia*. We created *de novo* assemblies from these datasets to obtain OP50 and *M. tb* genomes using metaSpades (Nurk et al., 2017). Similar to the ATAC-seq *Wolbachia* assemblies, we found higher read coverage on the bacterial contigs (Figure 8). These results demonstrate the bacterial sequence enrichment capabilities of ATAC-seq for bacteria with large genomes over 4 million bps.

## 4 Discussion

Sequencing of symbiont genomes is important in order to study complex co-evolution between species, including parasitic, mutualistic and obligate relationships (Moran et al., 2000; Wernegreen, 2002; McCutcheon and Moran, 2012). Here, we present a new use for ATAC-sequencing as a method of bacterial sequence enrichment for metagenome assembly. We used *B. malayi* containing the endosymbiotic bacteria *Wolbachia* as a proof of principal for *de novo* genome assembly. There is over 50-fold enrichment of bacterial sequences in our ATAC-seq datasets compared to that of the standard gDNA libraries. This is a result of the lack of histones in the bacteria. The Tn5 transposase is able to cut the bacterial DNA uniformly, whereas only a small fraction of eukaryotic chromatin is cut corresponding to regions where no nucleosomes are present. Therefore, only a small proportion of the *B. malayi* genome is sequenced, while the whole *Wolbachia* genome is sequenced at high levels. Future ATAC-seq datasets containing a mixture of prokaryotic and eukaryotic organisms will determine if this method can be used to quantify the ratio of bacterial cells and host cells present within a sample.

When mapped to the *B. malayi* nuclear chromosomes, the ATAC-seq reads pile up in peaks, corresponding to the accessible regions of the genome. The chromatin structure is unique across adult males and females, which may lead to their specialized gene expression. The peaks are highly enriched in gene promoters, with a proportion of 46.2%. This enrichment is higher than what is typically observed in mammals (Chung et al., 2018; Yan et al., 2020). Similar to *B. malayi*, nearly 50% of peaks fall in promoter regions in *Drosophila melanogaster* (Santiago et al., 2021; Dhall et al., 2023). There appears to be a smaller proportion of enhancers in nematodes and arthropods when compared to mammals, which may relate to their smaller genomes.

With the high proportion of reads mapping to the *Wolbachia* chromosome, we hypothesized that ATAC-seq could be an efficient sequencing method for *de novo* genome assembly of bacterial endosymbionts. We assembled genomes from both the ATAC-seq and gDNA libraries using metaSpades and performed quality assessment using Blobtools and QUAST (Gurevich et al., 2013; Laetsch and Blaxter, 2017; Nurk et al., 2017). The ATAC-seq assembly can be binned into *Wolbachia* and *B. malayi* contigs by read coverage alone, meaning a reference genome is not required to separate out the bacterial genome. This method can be useful when working with samples that contain unknown bacterial species. The ATAC-seq assembly is also higher quality than gDNA assembly across every metric measured, including contig number, N50, misassemblies, and nucleotide mismatches. The gDNA genome is over assembled, meaning that sequences are inappropriately incorporated into the assembly. This results in a longer length and an increased number of misassemblies. Additionally, the gDNA assembly has over 300 incorrect nucleotide changes compared to just one in the ATAC assembly. The gDNA assembly seems to be affected by *nuwt* sequences from the *B. malayi* nuclear genome, as these chromosomes have similar sequencing depth to the *w*Bm genome. Therefore, the assembler cannot determine which sequence is correct for the *Wolbachia* assembly, resulting in *B. malayi* sequence incorporation. The ATAC-seq assembly does not have this issue, as there is significantly more sequencing depth over the regions transferred as nuwts originating from the *Wolbachia*, resulting in incorporation of the correct bacterial sequences. ATAC-seq shows vast improvement on assembly with symbiotic species which have experienced lateral gene transfer. Additionally, with ATAC-seq assembly, as few as 1 million starting reads can be used, while maintaining high quality of the resulting genome, including total length, low contig number and a high proportion of assembled genes. This aspect of ATAC-seq allows for lower sequencing and computational costs when compared to standard deep sequencing methods.

One drawback to the ATAC-seq enrichment method is the use of short read sequencing. Longer reads cannot be used, as enrichment involves the cutting of the histone free DNA. Despite high read coverage across the entire *Wolbachia* genome, we still have some gaps between contigs, specifically in highly repetitive regions. Repeats significantly longer than our fragment sizes could not be assembled, regardless of coverage depth. While ATAC-seq can still be used for high quality *de novo* genome assembly, it will also be useful for bacterial population genomics or SNP analysis, where short read sequencing is commonly used in combination with a long-read based reference genome (Joseph and Read, 2010; Epstein et al., 2012; Cornejo et al., 2013). Optimization of the ATAC-seq protocol, such as enzyme dilution, or lower Tn5 temperature and incubation time, may allow for longer reads. The ATAC-seq libraries also show more of a GC bias than the gDNA libraries. Therefore, ATAC-seq appears to be more sensitive to PCR bias with increasing PCR cycles (Benjamini and Speed, 2012). In future experiments, a lower number of cycles can be used, as we obtained a high yield from the ATAC libraries using 10 cycles.

Many comparative genomics studies are from field and clinical samples, where the starting material is limited, and the bacteria are unculturable outside of the host. An additional advantage to ATAC-seq, beyond bacterial sequence enrichment, is low sample input requirements. ATAC-seq can be performed on as little as 500 total cells with less than a day of library preparation time (Grandi et al., 2022).

Finally, ATAC-seq bacterial enrichment can be used beyond *Wolbachia* genome assembly. Bacterial enrichment was found in two previously published ATAC-seq datasets in *C. elegans* and human DCs (Pacis et al., 2015; Daugherty et al., 2017). Over 80% of reads from the *C. elegans* library originated from their food source, OP50 *E.coli*. In human DCs ATAC-seq data, there was also a 50-fold enrichment of *M. tb* sequences, similar to what we observed in our *B. malayi* and *Wolbachia* dataset. We were able to perform *de novo* assembly on the *C. elegan* and human libraries, resulting in bacterial genomes around 4 million base pairs long. These results show the utility of ATAC-seq enrichment across a wide variety of samples containing both eukaryotes and bacteria.

## 5 Conclusion

ATAC-seq provides a novel enrichment method for unculturable endosymbiotic bacterial sequences and can improve metagenome assembled genomes. We assembled higher quality genomes using ATAC-seq compared to standard gDNA sequencing, with as few as 1 million starting reads. Lateral gene transfer is common between closely associated bacterial endosymbionts and their eukaryotic hosts. ATAC-seq is able to correctly assemble these sequences that have been transferred to the host nuclear genome, while standard gDNA sequencing results in incorporation of incorrect sequences due to similar sequencing coverage of the bacteria and host. Compared to other symbiont sequencing methods, ATAC-seq requires a very low amount of starting material and sequencing depth. One of the main benefits of this method is the ability to enrich, sequence, assemble and bin contigs from unknown bacterial species, as no reference genome is required. ATAC-seq bacterial sequence enrichment will be beneficial for studying the complex relationships between bacteria and eukaryotes in symbiosis, infectious disease, agriculture, and microbiome research.

## Data availability statement

The datasets presented in this study can be found in online repositories. The names of the repository/repositories and accession number(s) can be found at: https://www.ncbi.nlm.nih.gov/, PRJNA1043224 https://figshare.com/, doi:10.6084/m9.figshare.24703149.v1 https://figshare.com/, doi:10.6084/m9.figshare.24701145.v1 https://figshare.com/, doi:10.6084/m9.figshare.24702282.v1 https://figshare.com/, doi:10.6084/m9.figshare.24702858.v1.

## Author contributions

LC: Conceptualization, Formal analysis, Investigation, Methodology, Visualization, Writing – original draft, Writing – review & editing. JD: Funding acquisition, Supervision, Writing – review & editing. JF: Conceptualization, Funding acquisition, Supervision, Writing – review & editing.
