# Supplementary material for: Improved metagenome assemblies through selective enrichment of bacterial genomic DNA from eukaryotic host genomic DNA using ATAC-seq
Source: Front Microbiol. 2024 Feb 15;15:1352378. doi: 10.3389/fmicb.2024.1352378 (PMC10902005; doi:10.3389/fmicb.2024.1352378)
Supplement: Supplementary file 1 [file Presentation_1.PPTX]

## Slide 1
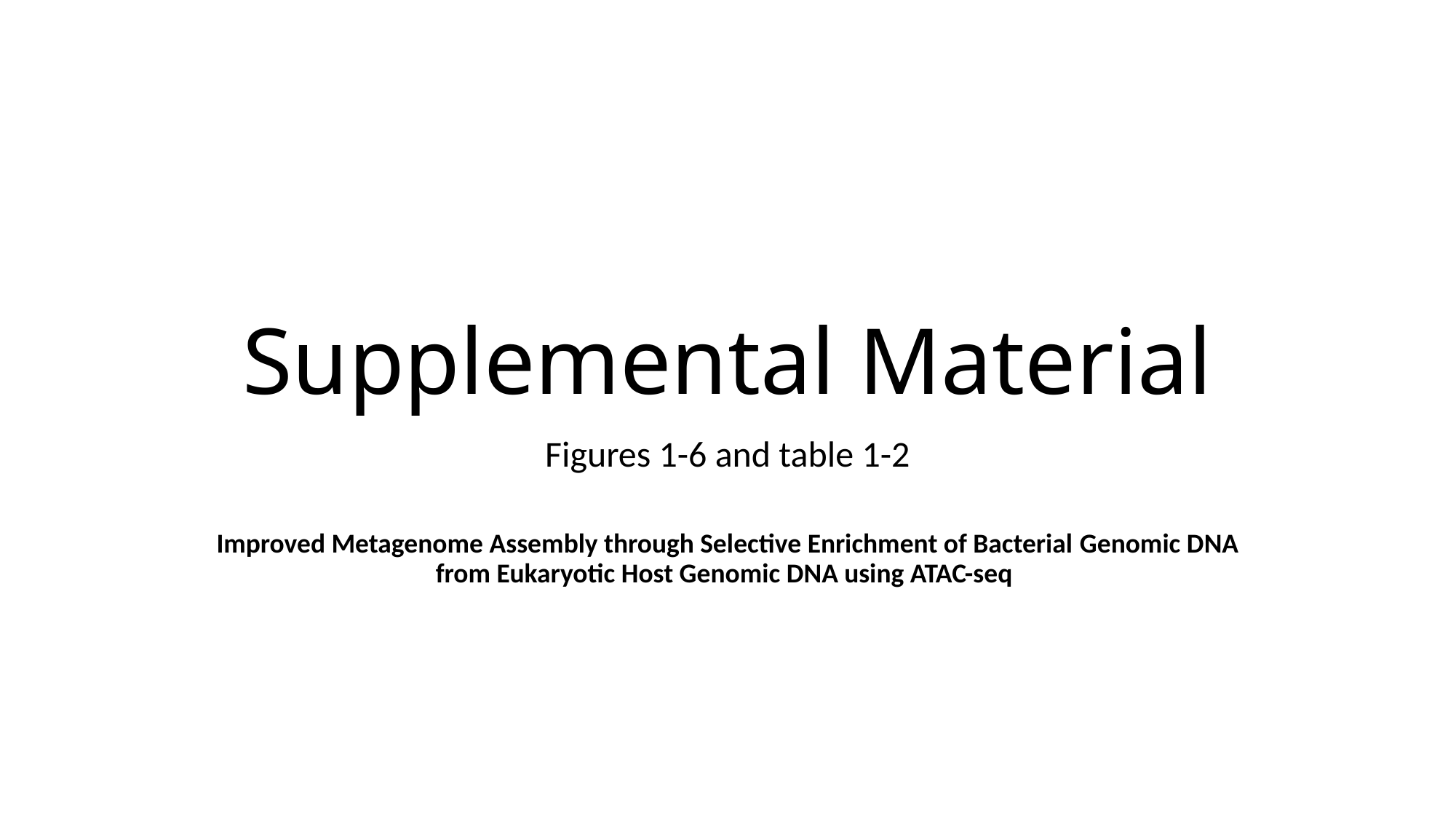

# Supplemental Material
Figures 1-6 and table 1-2
Improved Metagenome Assembly through Selective Enrichment of Bacterial Genomic DNA from Eukaryotic Host Genomic DNA using ATAC-seq

## Slide 2
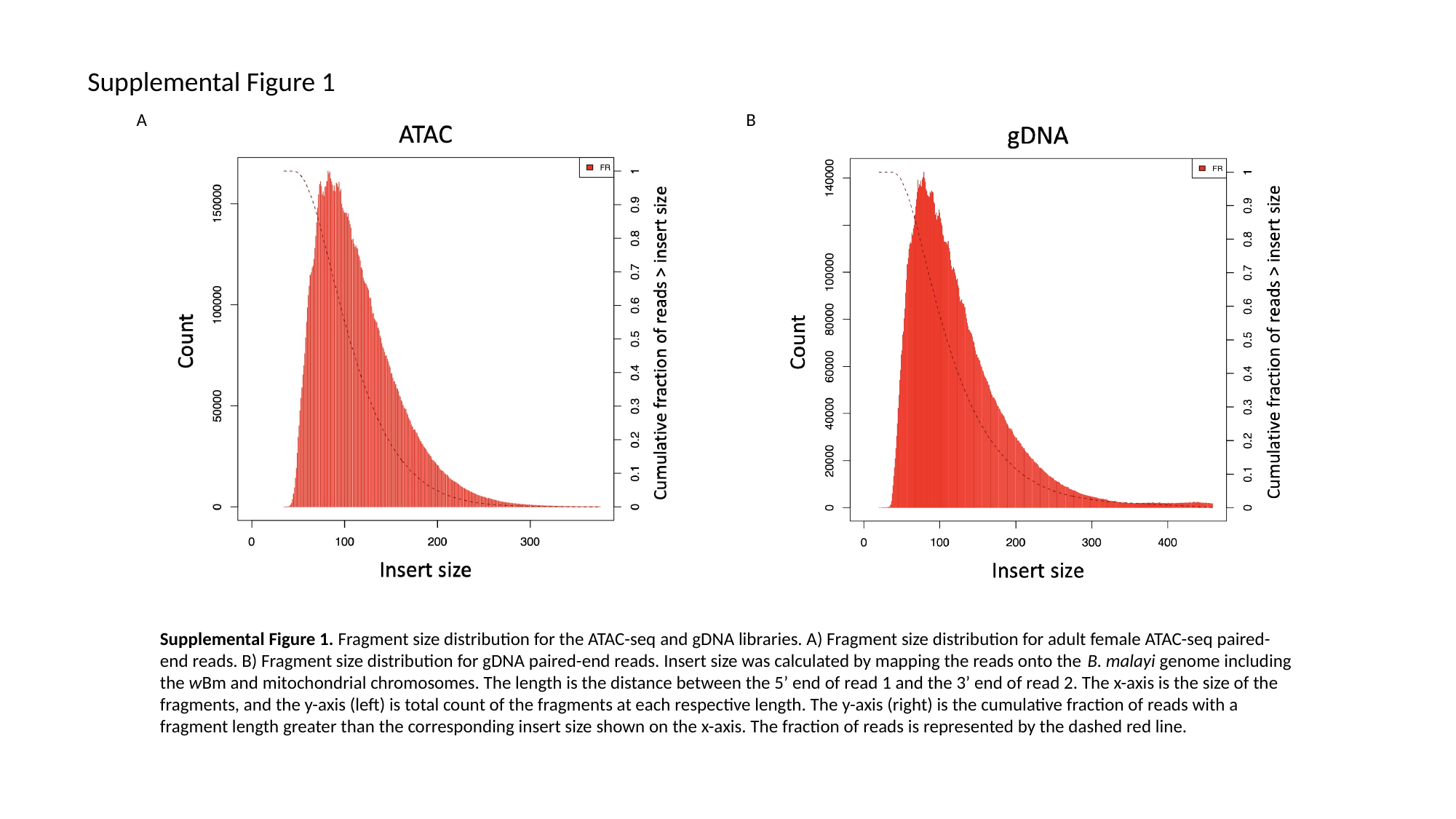

Supplemental Figure 1
A
B
Supplemental Figure 1. Fragment size distribution for the ATAC-seq and gDNA libraries. A) Fragment size distribution for adult female ATAC-seq paired-end reads. B) Fragment size distribution for gDNA paired-end reads. Insert size was calculated by mapping the reads onto the B. malayi genome including the wBm and mitochondrial chromosomes. The length is the distance between the 5’ end of read 1 and the 3’ end of read 2. The x-axis is the size of the fragments, and the y-axis (left) is total count of the fragments at each respective length. The y-axis (right) is the cumulative fraction of reads with a fragment length greater than the corresponding insert size shown on the x-axis. The fraction of reads is represented by the dashed red line.

## Slide 3
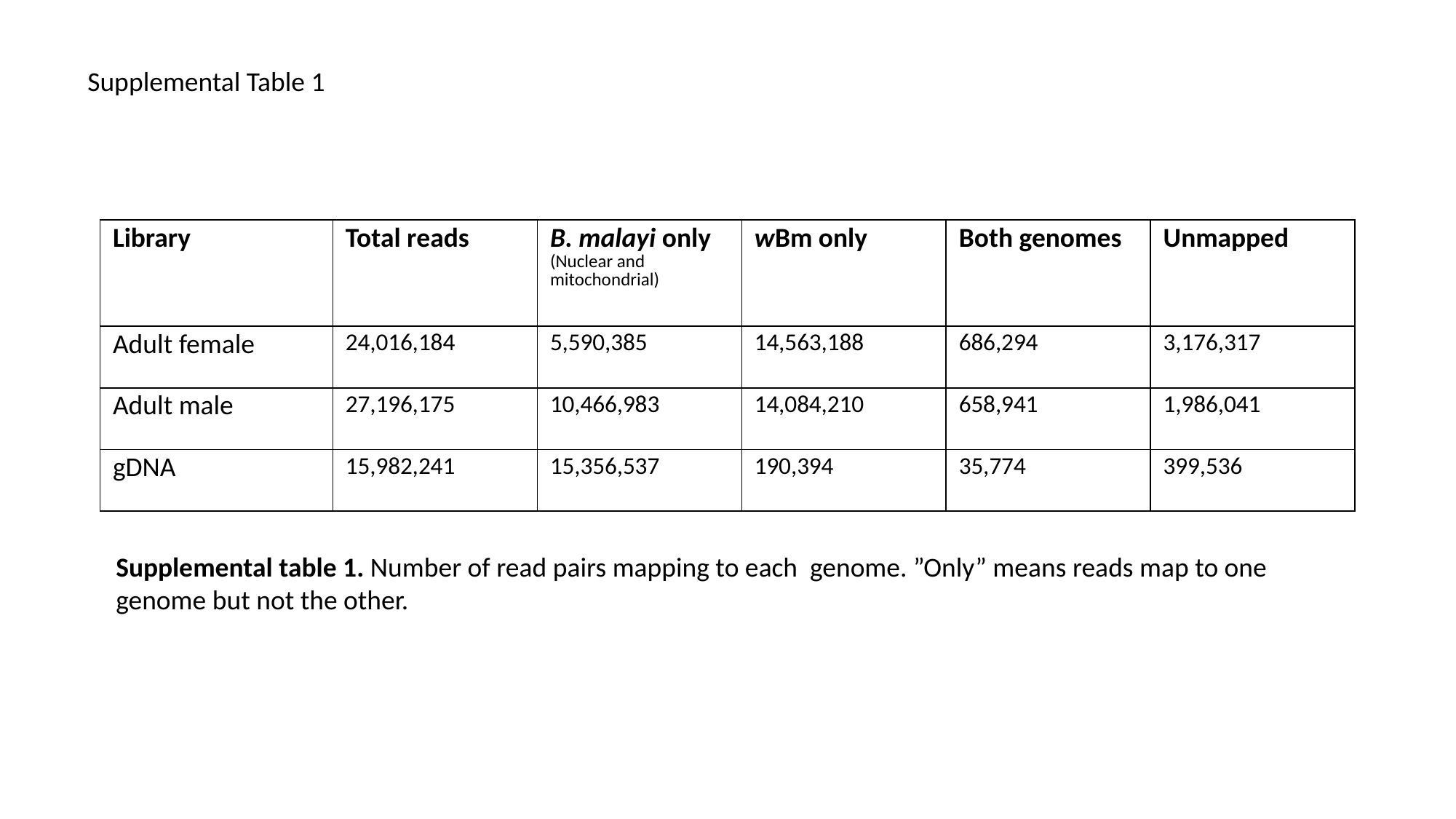

Supplemental Table 1
| Library | Total reads | B. malayi only (Nuclear and mitochondrial) | wBm only | Both genomes | Unmapped |
| --- | --- | --- | --- | --- | --- |
| Adult female | 24,016,184 | 5,590,385 | 14,563,188 | 686,294 | 3,176,317 |
| Adult male | 27,196,175 | 10,466,983 | 14,084,210 | 658,941 | 1,986,041 |
| gDNA | 15,982,241 | 15,356,537 | 190,394 | 35,774 | 399,536 |
Supplemental table 1. Number of read pairs mapping to each genome. ”Only” means reads map to one genome but not the other.

## Slide 4
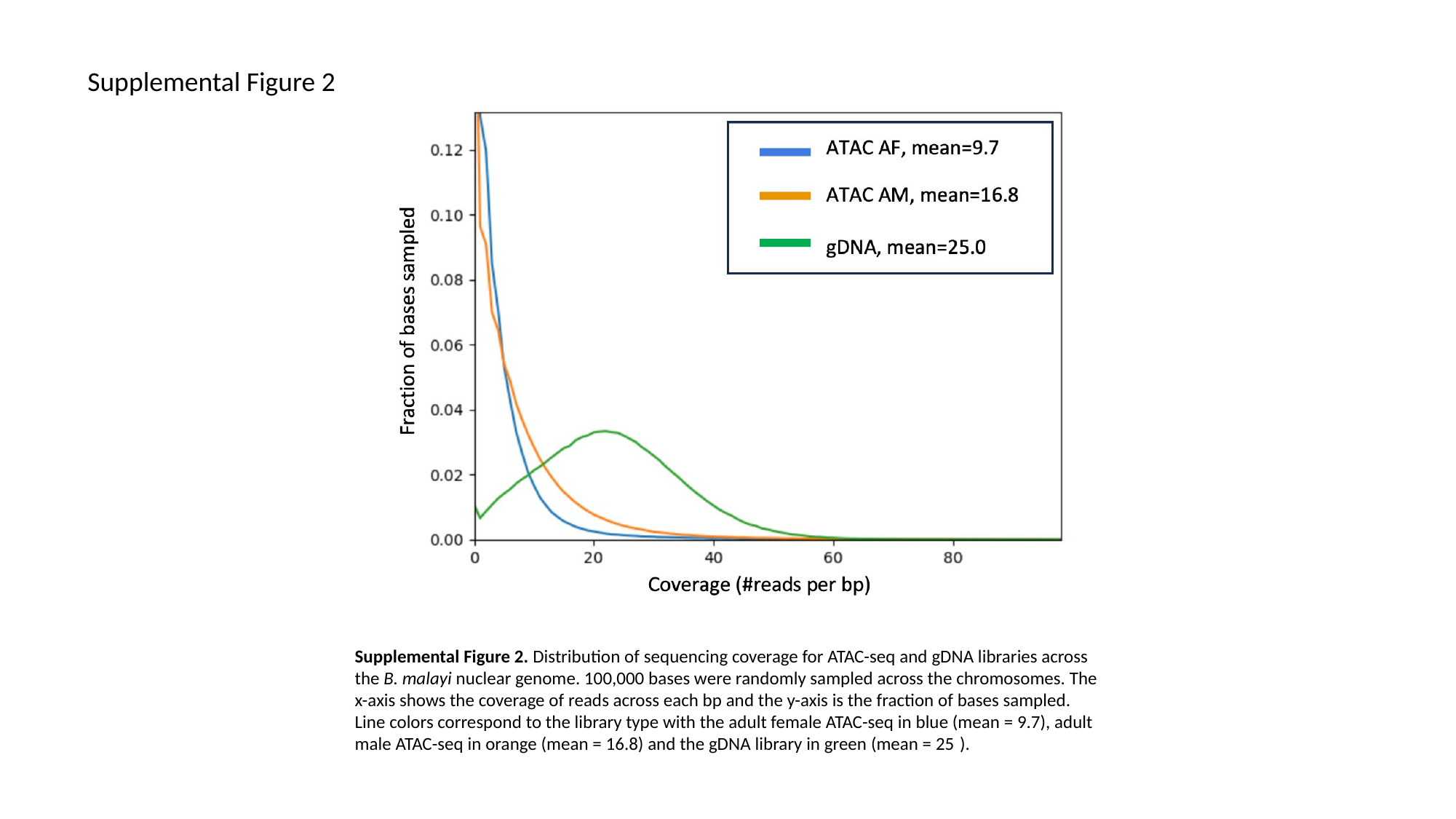

Supplemental Figure 2
Supplemental Figure 2. Distribution of sequencing coverage for ATAC-seq and gDNA libraries across the B. malayi nuclear genome. 100,000 bases were randomly sampled across the chromosomes. The x-axis shows the coverage of reads across each bp and the y-axis is the fraction of bases sampled. Line colors correspond to the library type with the adult female ATAC-seq in blue (mean = 9.7), adult male ATAC-seq in orange (mean = 16.8) and the gDNA library in green (mean = 25 ).

## Slide 5
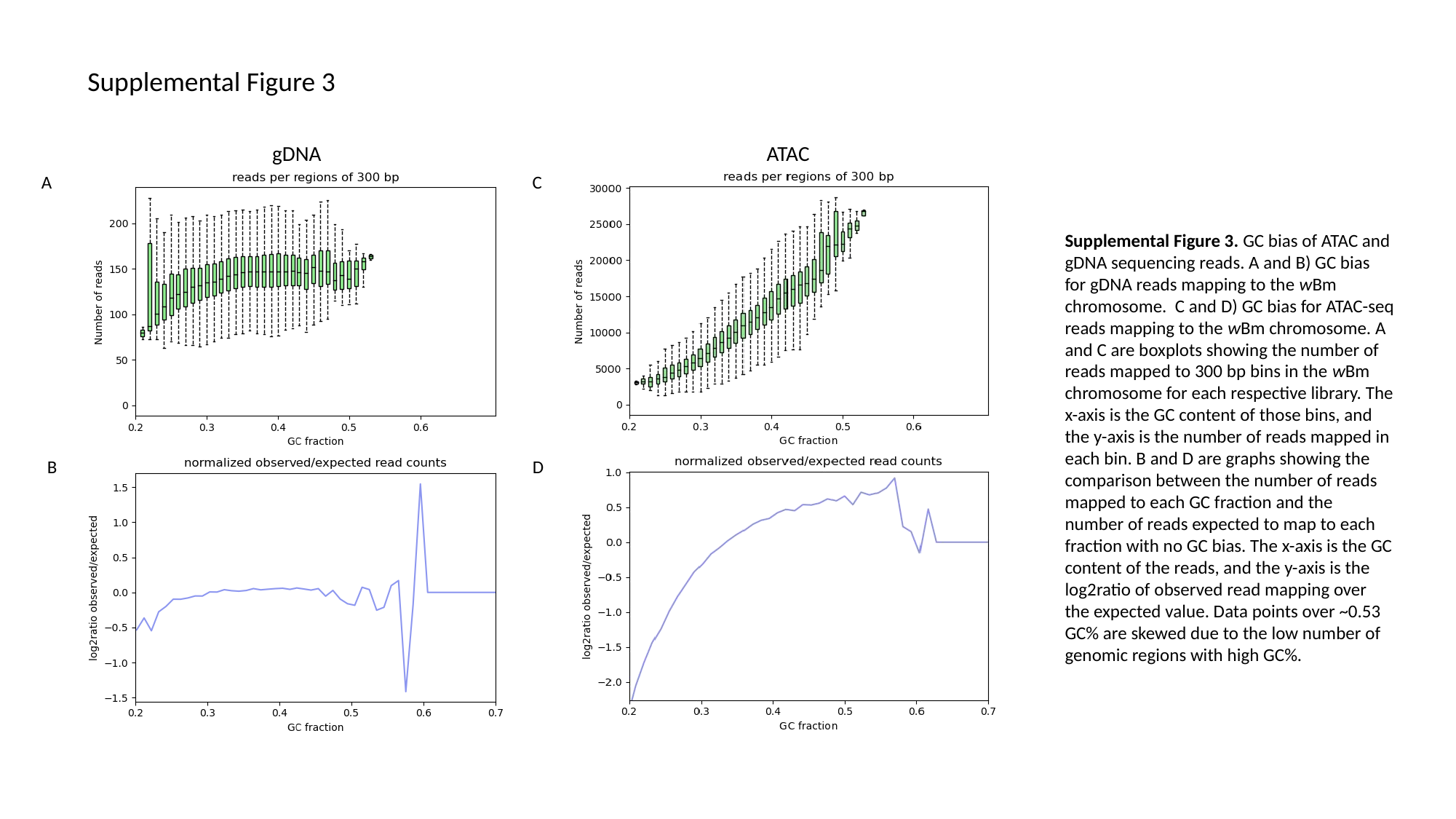

Supplemental Figure 3
ATAC
gDNA
C
A
Supplemental Figure 3. GC bias of ATAC and gDNA sequencing reads. A and B) GC bias for gDNA reads mapping to the wBm chromosome. C and D) GC bias for ATAC-seq reads mapping to the wBm chromosome. A and C are boxplots showing the number of reads mapped to 300 bp bins in the wBm chromosome for each respective library. The x-axis is the GC content of those bins, and the y-axis is the number of reads mapped in each bin. B and D are graphs showing the comparison between the number of reads mapped to each GC fraction and the number of reads expected to map to each fraction with no GC bias. The x-axis is the GC content of the reads, and the y-axis is the log2ratio of observed read mapping over the expected value. Data points over ~0.53 GC% are skewed due to the low number of genomic regions with high GC%.
D
B

## Slide 6
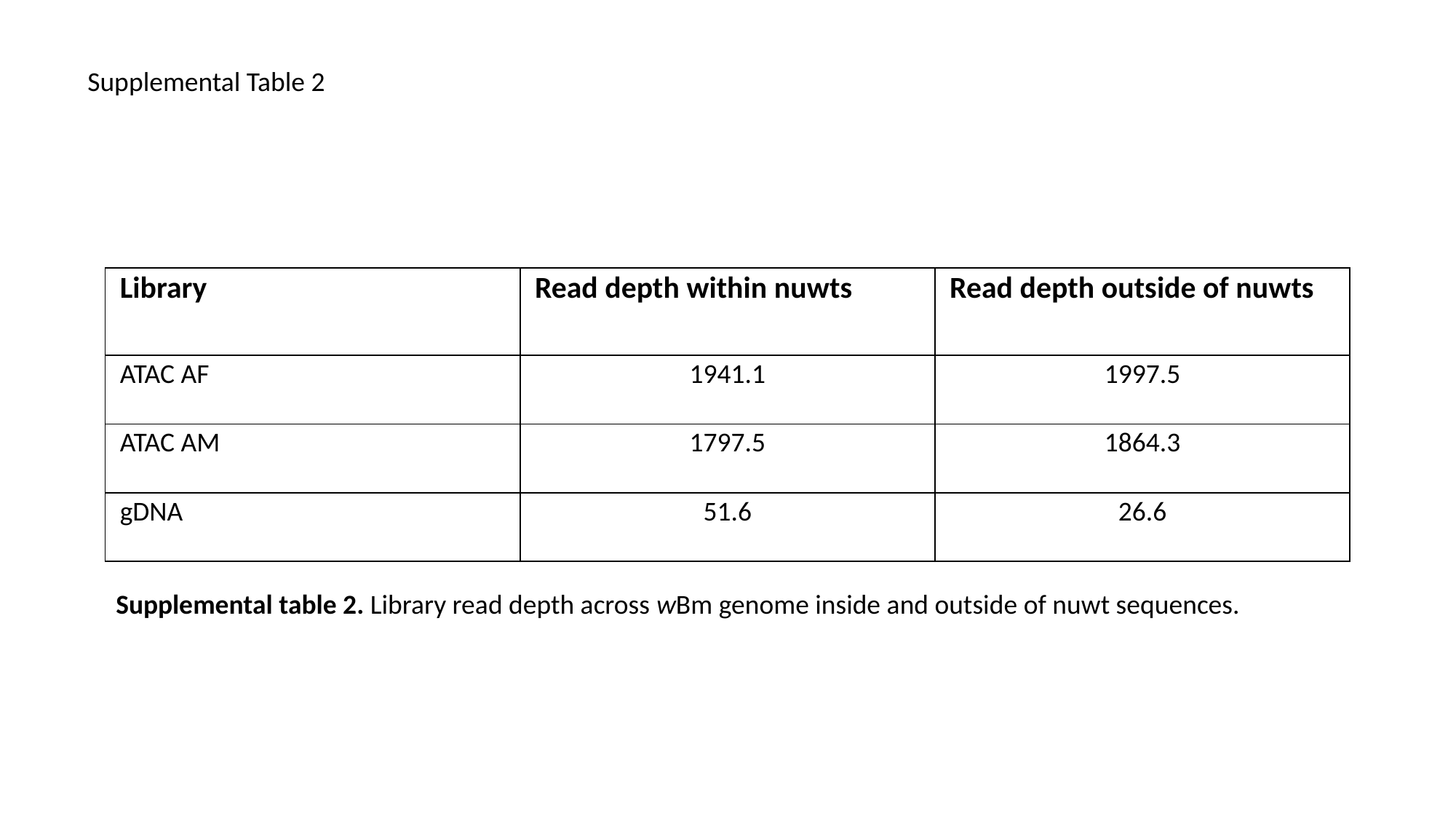

Supplemental Table 2
| Library | Read depth within nuwts | Read depth outside of nuwts |
| --- | --- | --- |
| ATAC AF | 1941.1 | 1997.5 |
| ATAC AM | 1797.5 | 1864.3 |
| gDNA | 51.6 | 26.6 |
Supplemental table 2. Library read depth across wBm genome inside and outside of nuwt sequences.

## Slide 7
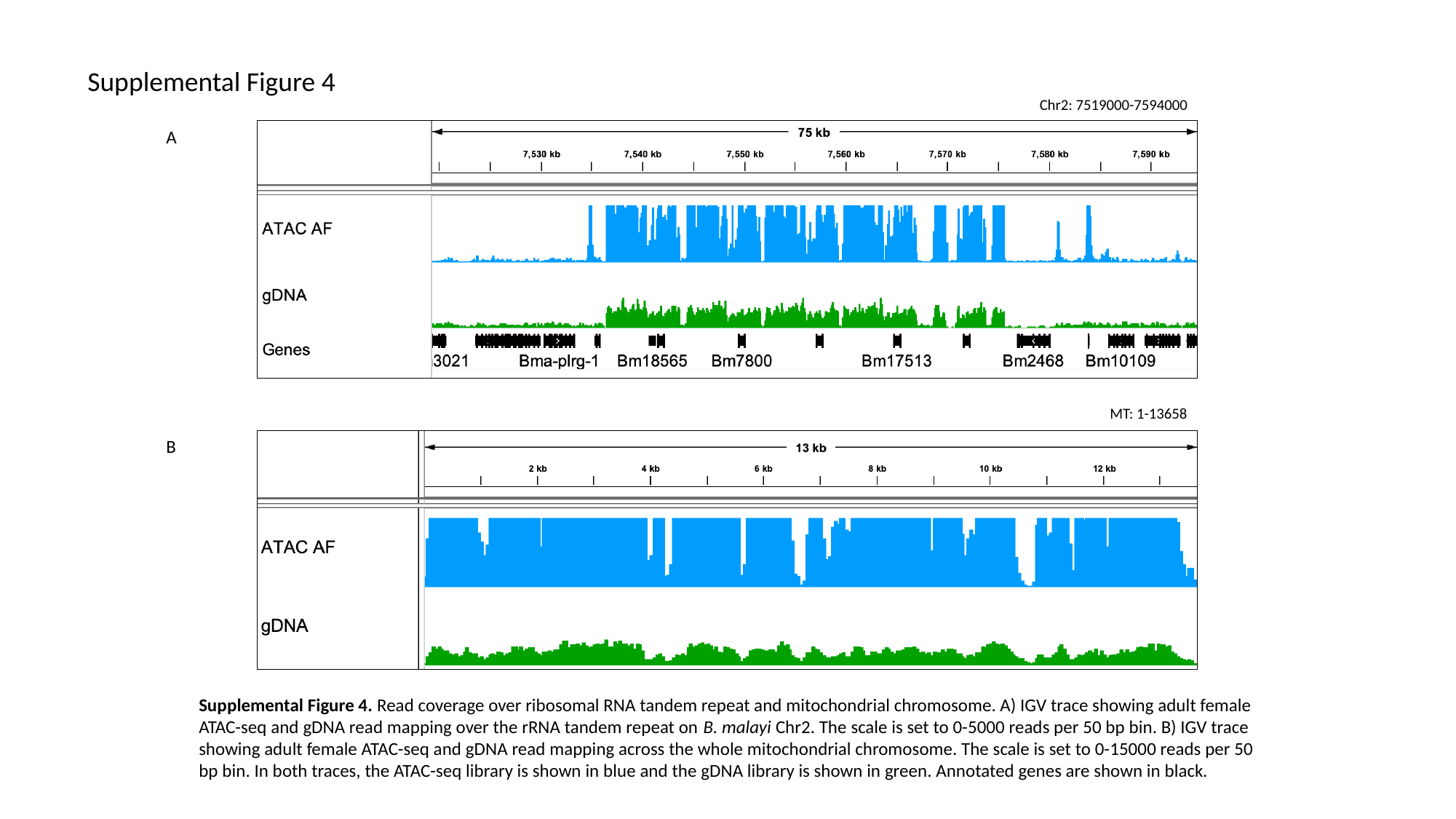

Supplemental Figure 4
Chr2: 7519000-7594000
A
MT: 1-13658
B
Supplemental Figure 4. Read coverage over ribosomal RNA tandem repeat and mitochondrial chromosome. A) IGV trace showing adult female ATAC-seq and gDNA read mapping over the rRNA tandem repeat on B. malayi Chr2. The scale is set to 0-5000 reads per 50 bp bin. B) IGV trace showing adult female ATAC-seq and gDNA read mapping across the whole mitochondrial chromosome. The scale is set to 0-15000 reads per 50 bp bin. In both traces, the ATAC-seq library is shown in blue and the gDNA library is shown in green. Annotated genes are shown in black.

## Slide 8
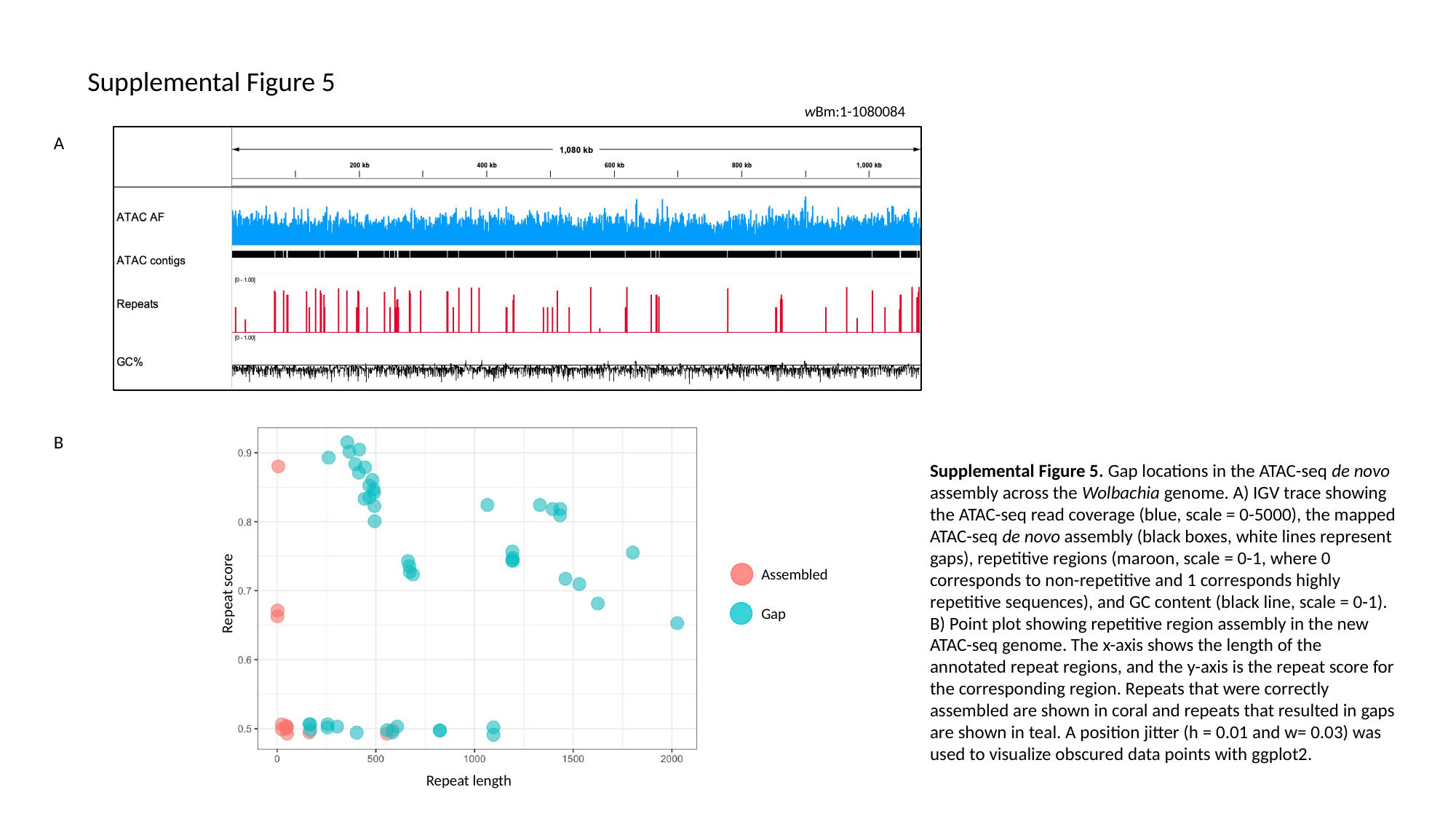

Supplemental Figure 5
wBm:1-1080084
A
Assembled
Gap
Repeat score
Repeat length
B
Supplemental Figure 5. Gap locations in the ATAC-seq de novo assembly across the Wolbachia genome. A) IGV trace showing the ATAC-seq read coverage (blue, scale = 0-5000), the mapped ATAC-seq de novo assembly (black boxes, white lines represent gaps), repetitive regions (maroon, scale = 0-1, where 0 corresponds to non-repetitive and 1 corresponds highly repetitive sequences), and GC content (black line, scale = 0-1). B) Point plot showing repetitive region assembly in the new ATAC-seq genome. The x-axis shows the length of the annotated repeat regions, and the y-axis is the repeat score for the corresponding region. Repeats that were correctly assembled are shown in coral and repeats that resulted in gaps are shown in teal. A position jitter (h = 0.01 and w= 0.03) was used to visualize obscured data points with ggplot2.

## Slide 9
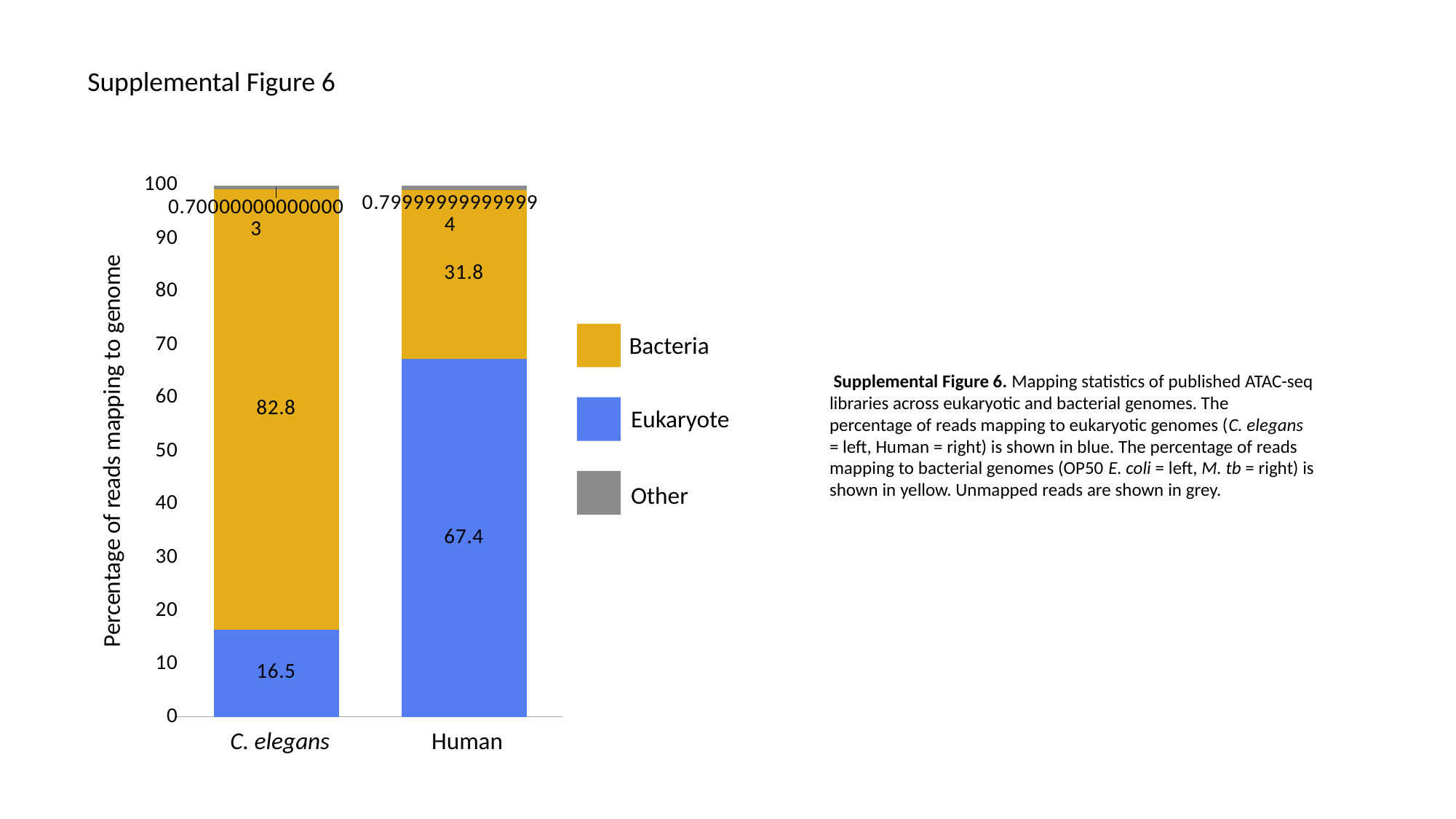

Supplemental Figure 6
### Chart
| Category | Host | Bacteria | Other |
|---|---|---|---|
| C. elegans | 16.5 | 82.8 | 0.7000000000000028 |
| Human | 67.4 | 31.8 | 0.7999999999999936 |
Bacteria
 Supplemental Figure 6. Mapping statistics of published ATAC-seq libraries across eukaryotic and bacterial genomes. The percentage of reads mapping to eukaryotic genomes (C. elegans = left, Human = right) is shown in blue. The percentage of reads mapping to bacterial genomes (OP50 E. coli = left, M. tb = right) is shown in yellow. Unmapped reads are shown in grey.
Eukaryote
Other
Human
C. elegans
